# Development and content validity of the Abilitator: a self-report questionnaire on work ability and functioning aimed at the population in a weak labour market position

**DOI:** 10.1186/s12889-020-8391-8

**Published:** 2020-03-14

**Authors:** Miia Wikström, Heidi Anttila, Minna Savinainen, Anne Kouvonen, Matti Joensuu

**Affiliations:** 1 Finnish Institute of Occupational Health, Helsinki, Finland; 2 Finnish Institute for Health and Welfare, Helsinki, Finland; 3 Finnish Institute of Occupational Health, Tampere, Finland; 4 Faculty of Social Sciences, University of Helsinki, Helsinki, Finland; 5 Research Institute of Psychology, SWPSSWPS University of Social Sciences and Humanities, Wroclaw, Poland; 6 UKCRC Centre of Excellence for Public Health (Northern Ireland), Queen’s University Belfast, Belfast, UK

**Keywords:** Work ability, Functioning, Health, Well-being, Self-report, Unemployment, Employability, Inclusion, ICF, Content validity

## Abstract

**Background:**

The unemployed have lower work ability and poorer health than the employed. This situation deteriorates when unemployment continues. The long-term unemployed often have co-morbidities and face many other challenges. This increases the need for a multidimensional assessment of work ability and functioning in different service settings. In this study, we describe the development and analyse the content validity of the Abilitator, a self-report questionnaire on work ability and functioning for those in a weak labour market position.

**Methods:**

The Abilitator was developed in 2014–2017. Its construct was assessed by members of academic expert panels (*n* = 30), practical expert panels of professionals (*n* = 700) and target group clients (*n* = 28). The structure and the content of the questionnaire was co-developed in 29 workshops and adjusted twice based on the expert panels’ feedback. The Abilitator was also implemented among target group clients (*n* = 3360) in different services and projects. During its development the Abilitator was linked to the International Classification of Functioning, Disability and Health (ICF). The content validation process followed the guidelines recommended by the Consensus-based Standards for the selection of health Measurement Instruments (COSMIN) panel.

**Results:**

The construct of the Abilitator combines the multidimensional and biopsychosocial models of work ability and functioning. It also includes aspects of social inclusion and employability. It evaluates social, psychological, cognitive and physical functioning, and the ability to cope with everyday life. The content of these concepts was validated by the academic and practical expert panels. The Abilitator’s 79 ICF codes covered 57% of the Generic, 77% of the Brief Vocational Rehabilitation, and 8% of the Minimal Environmental ICF Core Sets. When compared with the Work Ability Index (WAI) and the World Health Organization Disability Assessment Schedule (WHODAS 2.0), the direct equivalences of the ICF codes were 36 and 44%, respectively.

**Conclusion:**

The Abilitator sufficiently comprehensively covers the relevant aspects to enable the assessment of the overall work ability and functioning of the population in a weak labour market position.

## Background

It is well established that unemployment is associated with a lower educational level and poorer health and well-being [1–6]. In 2017 in the European Union (EU), the unemployment rate was 7.6% and of those who were unemployed, 45.1% were long-term unemployed [7]. Prolonged unemployment may lead to social exclusion, marginalisation, and inequality in working life [4, 8–10]. The evidence suggests that reducing unemployment would lead to improved quality of life and health outcomes and should be a priority [11].

Work ability can be defined as a combination of health, functioning, basic standard competence and the relevant occupational virtues required for managing reasonable work tasks in an acceptable environment [12, 13]. It is associated with all factors of working life: the individual, the workplace, the immediate social environment, and the society [13]. It can be used to specify the expectations of employees in terms of the competence needed for different kinds of work, in disease prevention and health promotion and as an instrument to determine the degree and type of rehabilitation needed by individuals. It is also a central legal concept regulating sickness and social insurance policies [12].

Functioning is closely related to health and comprises a psychological, social, physical and cognitive dimension [14, 15]. Psychological functioning is the ability to feel, experience, form perceptions of oneself and the surrounding world, plan life, find solutions, and make decisions [16]. Social functioning is manifested through one’s role as an actor with and among others, interaction with social networks, social activities and participation, as well as experiences of coexistence and inclusion [17]. Inclusion means that a person feels they are a significant part of an entity with others. Inclusion is a process that can be observed through material, spiritual, social, and physical dimensions and can be viewed from a variety of perspectives, such as education or work [18]. Physical functioning includes the ability to physically perform everyday basic activities and meaningful leisure activities, as well as to work and study [19]. Cognitive functioning is the mental function related to the reception, processing, preservation, and use of knowledge [20].

The unemployed have lower work ability than those who are employed [21–24]. Contemporary working life places new and rapidly increasing demands on individuals’ work ability, functioning and employability [25]. These pressures accumulate, especially among those who are in a weaker labour market position to begin with. This is a heterogenous group of people who repeatedly or continuously have difficulties gaining employment: for example, those with less education and fewer skills, disabilities, or chronic health problems, those experiencing long-term unemployment and those with migrant backgrounds [26–29]. These people may particularly benefit from individually targeted actions to improve their opportunities for participation and employment [30, 31].

The relationship between unemployment and health has shown to be bi-directional: poor health can cause unemployment and unemployment can cause poor health [3, 4]. In addition to lower work ability, the unemployed have lower self-rated health and life satisfaction than those who are employed [1, 24, 32]. It has been estimated that in long-term unemployment, financial difficulties and a low educational level explain half of the factors related to low self-rated work ability [24]. Mental disorders, neurological disabilities, musculoskeletal problems and substance abuse challenges have also been associated with low work ability [27]. In addition, the long-term unemployed often have several simultaneous health impairments, which makes the individuality and multidimensionality of the assessment of their work ability and functioning even more difficult [26, 27].

Most Western European welfare states have a vast array of public, private or third sector services available to support and promote the health, rehabilitation, social well-being, education and employment of the working-age population. These services often need to assess work ability and functioning individually. It has been suggested that the resources of, for example, health services should focus more on those whose perceived health and work ability has started to decline [22, 33]. It has also been recognised that many work ability limitations go unnoticed by these services, resulting in unused opportunities for rehabilitation [2, 34, 35]. In terms of work ability, the unemployed can be split into three groups: 1) those with good work ability, 2) those unable to work, and 3) those whose work ability can be restored with adequate rehabilitation [27]. These groups vary greatly in their needs for support [34]. To direct resources appropriately, new approaches are needed to assess work ability and functioning more individually.

The challenges to assess work ability and functioning of those in a weak labour market position can be viewed from four operational angles. First, the individuals may not recognise the challenges in their work ability and functioning; they may find it difficult to express their own views of their life situation, perceived abilities and challenges. Factors such as long-term unemployment can lead to reduced self-esteem and feelings of shame [36], less trust in services, and a lower perceived ability to cope with working life [37]. We may need neutral, positively structured self-report instruments of work ability and functioning.

Second, a few generic, feasible and validated self-report instruments exist for the multidimensional assessment of work ability and functioning among the unemployed [38, 39]. Current instruments are designed for professionals in vocational rehabilitation or occupational health services; instruments are needed that are easy to interpret without medical or other specific professional training, as the occupational backgrounds of the personnel in these services can vary.

Third, these services’ use of assessment-based procedures and unified ways of encountering the unemployed individually are in their infancy [26]. Such processes are required for co-operation with service clients to create more focused service plans. These assessment procedures should simultaneously support the individual’s agency.

Fourth, organisations providing employment and other welfare services should assess the effectiveness of their services [40]. Reliable information on clients’ needs and the changes that take place during the service process is needed for planning the allocation of resources. Patient-Reported Outcome Measures (PROMs) have been suggested as a good measure of services’ impact on clients’ well-being and their ability to play an active role in society. PROMs are questionnaires for clients of different services on their health, functioning and health-related quality of life. The information collected from these subjective reports enable following the clients’ progress and facilitating communication between professionals and clients and help improve the quality of services [41].

To meet these needs, we developed the Abilitator – a generic, multidimensional instrument for self-reporting of work ability and functioning among the population in a weak labour market position. It is a digital questionnaire that analyses responses and produces individual written feedback with suggestions for further actions to maintain or improve work ability and functioning. The main purpose of the Abilitator is to help individuals (clients) identify their strengths and challenges in terms of their work ability and functioning, thus improving their awareness of their life situation when accessing different services. The self-report aims to provide the client a structured basis for individual goal setting in a subsequent dialogue with professionals in health, rehabilitation, social services, education and employment services. Its purpose is to help the professionals work together with the clients to implement the most suitable measures and interventions to reach the set goals.

The aim of this study is to describe the development and assess the content validity of the Abilitator and its alignment with the International Classification of Functioning, Disability and Health (ICF) to assess the overall work ability and functioning of the population in a weak labour market position.

## Methods

The Abilitator was developed at the Finnish Institute of Occupational Health (FIOH) by the Social Inclusion and the Change of One’s Work Ability and Capacity (Solmu) project, which is a national co-ordination project funded by the European Social Fund (ESF) Priority 5 programme (2014–2020). The goal of this ‘Social inclusion and combating poverty’ ESF programme is to improve the work ability and functioning of people outside working life. The aim of Solmu was to co-develop feasible procedures and a method for evaluating changes and improvements in the participants’ work ability and functioning during the projects funded by the ESF Priority 5 programme.

### Sample

The clients participating in services provided by the ESF Priority 5 projects represented the target group sample. They were mainly of working age, had been unemployed for several years, and faced various problems related to their health, lifestyles and life situations. They participated in the projects voluntarily to improve their work ability, functioning and employment opportunities. On average they were 40 years of age, had been unemployed from 3 to 7 years and had little post-compulsory education.

The essential development of the Abilitator was carried out by an internal group of experts (*n* = 8) from FIOH representing medical, health, sport, behavioural, and social sciences. One member of this group was from THL. The first external expert panel included academic specialists (*n* = 30) from the fields of work ability, functioning and social inclusion. The second external expert panel included both professionals and target group clients, bringing expertise through experience to the development. The professionals (*n* = 700) had varying lengths of work experience with the target population, and their occupational backgrounds ranged from university research scientists to social workers and sports coaches. They mostly worked in ESF Priority 5 projects. The target group experts (*n* = 28) were clients who received the services in the ESF Priority 5 projects.

### Development

**Fig. 1:**
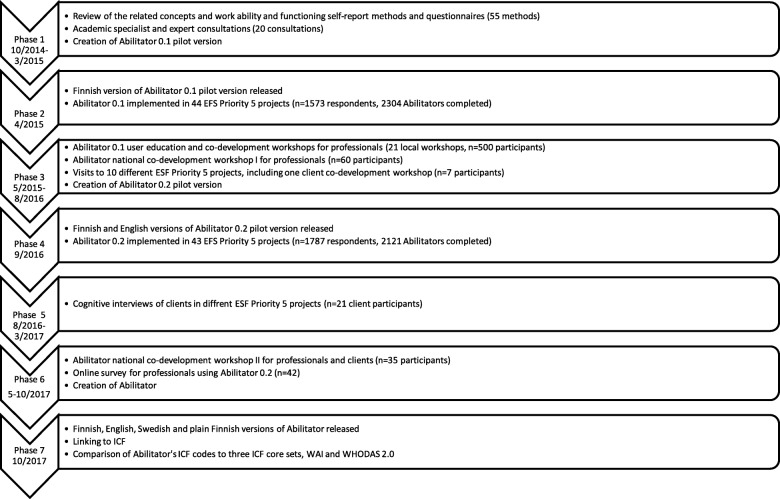
Development of the Abilitator [modified from 43]

The development of the Abilitator progressed through seven phases between 2014 and 2017 (Fig. 1). In Phase 1, we reviewed the related theoretical models and the existing PROMs of work ability and functioning used in Finland and more widely in the EU. We searched Google Scholar and PubMed using terms such as work ability, functioning, functional capacity, model, method, concept, theoretical, self-report, self-assessment, and questionnaire. We also identified self-report instruments from the Finnish TOIMIA network’s database, which contains guidelines for the measurement of functioning and evaluations of these measures [42]. The inclusion criteria for the instruments were their proven reliability, validity and wide use in previous research of the working-age population. Further inclusion criteria were free access, availability in the Finnish language and different occupational groups being able to use the instrument safely. The first version (0.1) of the Abilitator was formed based on the review and co-development with academic experts (*n* = 20).

After Abilitator 0.1 was implemented in 44 ESF Priority 5 projects in Phase 2, we co-developed it with professionals, academic experts and clients to form the second version (0.2) (Phase 3). The professionals’ (*n* = 600) experiences of and suggestions regarding the content of Abilitator 0.1 were collected in 22 local or national co-development workshops. Each workshop covered the content of the whole questionnaire in a similar manner to determine: 1) whether each question was relevant for the target group, 2) whether each question was formulated in a way that was appropriate for the target group, and 3) what kind of alterations should be made to each question for them to better suit the target groups’ needs or situations. Similar feedback was gathered from the professionals during 10 visits to different EFS Priority 5 projects. One group of clients (*n* = 7) also suggested question alterations and academic experts (*n* = 15) gave their input in separate encounters at this stage.

In Phase 4, Abilitator 0.2 was implemented in the ESF Priority 5 projects. After this, cognitive interviews were conducted with clients (*n* = 21) participating in five different national or local ESF Priority 5 projects (Phase 5) [43]. The interviewed groups had good geographical, gender and target group coverage. The aim of the interviews was to obtain information on how the respondents had processed and interpreted the questions of Abilitator 0.2. The interviews used a four-step question-answer process [44] related to the format, feasibility and comprehension of the questions. All the interviews were conducted by two interviewers and progressed following the same pattern. Each interview was recorded and transcribed.

In Phase 6, we sent an online survey to all the professionals (*n* = 144) using Abilitator 0.2 to collect additional feedback on the content, feasibility and format of the instrument. We also ran a second national co-development workshop with the professionals (*n* = 35). We used the information and feedback gained from the survey (*n* = 42), the workshop and the cognitive interviews to steer the development of Abilitator 0.2’s content and layout. The feedback was again systematically gathered in written format and reviewed by the internal group of experts. The suggestions were grouped into similar feedback units and the decisions regarding changes to the questionnaire were made in the internal expert group’s consensus meetings. At the end of Phase 6, the third version, i.e. Abilitator was ready. During each phase of the development, the professionals were given training and support materials on how to use the Abilitator with their clients and how to interpret the results.

When the content development was complete, the Abilitator was linked to the ICF (Phase 7). The main purpose of this was to translate the instrument’s content into the internationally unified and consistent language of human functioning, which can be used as a reference for comparing health information. This linking was conducted in co-operation with the national ICF concept working group and followed the updated linking rules [45]. It was first conducted by two research scientists separately and consensus was reached in two separate sessions with three other ICF experts. The second purpose was to position the Abilitator among the ICF-linked self-report instruments measuring work ability and functioning, and to compare the Abilitator’s ICF codes with the three ICF Core Sets most relevant to the target population; the generic set (7 codes), the brief vocational rehabilitation set (13 codes) and the minimal environmental set (12 codes) [46].

The Abilitator’s ICF codes were further compared with two validated, central self-report instruments: the Work Ability Index (WAI) (14 codes) and the World Health Organisation Disability Assessment Schedule (WHODAS 2.0) (27 codes). The WAI is used in occupational health services and research to assess employee work ability in health examinations and workplace surveys [39]. It is developed by FIOH to help define the necessary actions for maintaining and promoting work ability. WHODAS 2.0 is a generic assessment instrument that provides a standardised method for measuring health and disability across cultures. It was developed from a comprehensive set of ICF items that are sufficiently reliable for measuring activities and participation [47].

### Content validation

The content validation process of the Abilitator followed the guidelines of COSMIN [48, 49]. It was split into five phases: 1) definition of the construct to be measured and specification of the situation in which the instrument is used, 2) expert panels’ assessment of the instrument’s content during the development process, 3) consideration and provision of information on the instrument’s content, 4) assessment of whether the instrument’s content corresponded to the construct, and 5) assessment of whether the instrument’s construct corresponded to the ICF framework of functioning and relevant ICF Core Sets and other instruments measuring the same construct. Phase 2 included the evaluation of face validity, which is the degree to which the measurement instrument seems to be an adequate reflection of the measured construct [49].

## Results

### Specification of measured construct and context of use

The literature review conducted in Phase 1 (Fig. 1) found eight different theoretical models for work ability [50] and two models for functioning. In the bio-medical model of work ability, an existing illness, impediment or disability determines a person’s attributes and qualities as a worker [13, 51, 52]. In the balance model, work ability is the equilibrium between the individual and work-related factors [51, 53]. The psychosocial model emphasises the psychological and psychosocial factors connected to work participation and return to work [54–57]. In the multi-dimensional models and the bio-psycho-social models, work ability is a holistic, comprehensive entity in which individual resources and work-related factors are combined by the operational environment and social support [13, 14, 27, 58]. In the employability model, work ability combines all the individual and societal actions that help a person become employed, stay employed and advance their career [59, 60]. According to the model emphasising the integration of the individual at the workplace, the concept of work ability is based on continuous change in work and work organisation [61, 62]. Work ability can also be considered a social construct that is constituted by and differs between different societies and systems [63].

As with the concept of work ability, biomedical and biopsychosocial models have been used to describe functioning and health [58]. An internationally accepted way of structuring the concept of functioning is ICF [15], the framework of which provides a standard language and multi-purpose classification of disability and health [64]. Functioning is a collective umbrella term of the ICF that describes a person’s body structures and functions and their capacity to perform daily activities in the environment in which they live. The ICF is a biopsychosocial model that combines the biomedical, social and environmental aspects of human functioning, health and disability [14, 65]. It can be used as an instrument to collect comparable data to support evidence-based decision-making in health and health-related sectors. WHO and the ICF Research Branch have created Core Sets of ICF which the essential relevant categories for specific health conditions and health care contexts [46].

The ICF framework reflects six different aspects of health and disability: health condition, body structure and body function, activity, participation, environmental factors, and personal factors [66]. Diseases or disorders, i.e. health conditions, are included in the conceptual model of health, but are classified in the International Classification of Diseases and Related Health Problems (ICD) [67]. Functioning should be understood as a continuum ranging from completely able (non-problematic) to completely disabled (problematic) and is the result of complex multifactorial interaction between the six components [68].

#### Concepts of work ability and functioning in the Abilitator

The Abilitator is based on the multidimensional model of work ability [13] as this model describes both individual resources and the operational environment. We chose the ICF biopsychosocial model [14] for functioning because it is widely accepted in situations of multiple and long-term impairments of health [55].

The selected multi-dimensional work ability model is called the House of Work Ability [13, 69]. It has four levels that depict the relationship between individual resources, work-related demands, and the social and operational environments that affect both individual resources and working life. The three lower levels of the model describe individual resources such as health and functioning, competence and work experience, values, attitudes, and motivation. The top level is the level of work and includes factors related to work, working conditions, work community and leadership. Individual work ability is created by the balance between all the levels of the house, which are also significantly affected by social networks, communities and environments outside the workplace [13, 70, 71]. The Abilitator does not cover the top level, because those in a weak labour market position are to a large extent without employment.

The ICF biopsychosocial model of functioning [15, 58] sees operational constraints as a mismatch between the health of a person and the requirements of their life situation. To minimise this disparity, the impact of environmental and individual factors must also be considered in addition to the person’s health-related factors. These include available support and services, work situation, family, hobbies, motivation, and religion [72, 73].

**Fig. 2:**
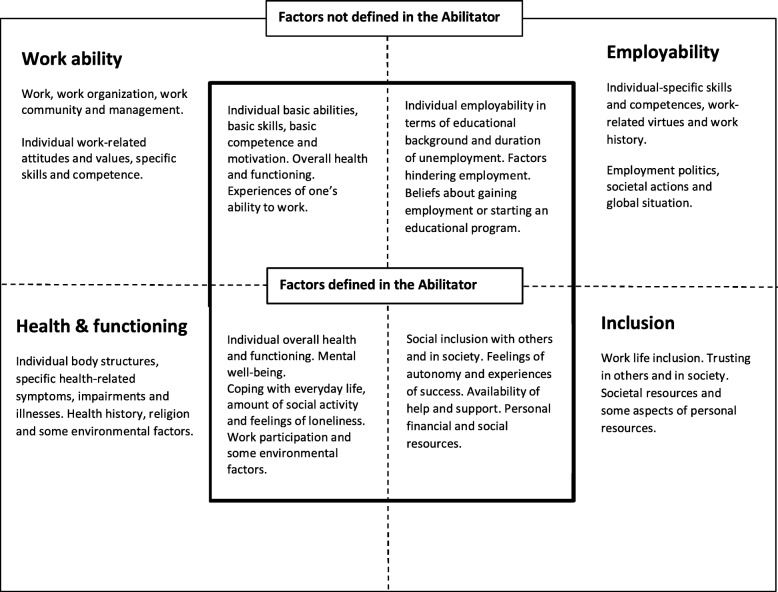
Construct of the Abilitator, consisting of concepts of work ability, health and functioning, inclusion and employability. The inner square features the factors of each concept that are defined in the Abilitator and the outer square those that were excluded. The dotted lines reflect the overlapping of the four concepts

The construct of the Abilitator can be further described using a framework of four central and partly overlapping concepts that can be linked to the population in a weak labour market position. These concepts are: 1) work ability [12, 13, 69], 2) health and functioning [15], 3) inclusion [18] and 4) employability [27, 60]. They include a variety of factors, some of which are defined in the Abilitator and some not, as shown in Fig. 2.

#### Specification of the Abilitator’s context of use

The Abilitator was developed to be suitable for individual and multidimensional self-assessment of the work ability and functioning of the population in a weak labour market position. The use contexts in the ESF projects included: to assess the service clients’ situations individually, to set goals, to design the best service plans to reach the set goals, and to make changes in work ability and functioning apparent to both clients and professional. This information was further used to analyse the effect of the different actions on larger groups of service clients taking part in ESF Priority 5 projects.

### Utilisation of expert panels in co-development and assessment of content

An example of the expert panels’ influence on the Abilitator content are questions D8 and D9 [Additional file 1]. Abilitator 0.1 contained two items for screening depression in primary care [74]. During Phase 3, systematic negative feedback from the target group, the professionals and the academic experts led to the removal of these items. The questions were considered too diagnostic to be used by professionals, too invasive to be answered by the respondents, and too difficult to evaluate in the context of their use. However, issues such as taking the initiative in everyday activities were still considered important. Therefore, the group of experts formulated two completely new items, D8 and D9. These questions were added to Abilitator 0.2 [Additional file 1] and the practical group of experts assessed their feasibility during Phases 5 and 6. Due to the systematically positive feedback received, Questions D8 and D9 of the Abilitator remained unchanged.

Abilitator 0.2 contained 76 questions and the online version also offered personal feedback. The content and format of this feedback was developed with the external expert panels along with the content of Abilitator 0.2. The internal expert group decided not to include all the questions in the feedback the Abilitator 0.2 gives the respondent because one single answer is not always enough to make meaningful assumptions about the respondent’s situation. However, the interpretations of all the questions were analysed for the professionals in the Abilitator user manual.

Based on the literature review on instruments in Phase 1 the internal group of experts created the structure and content of Abilitator 0.1 from pre-existing questionnaires and some newly formed questions into one self-report questionnaire. The literature review identified 55 self-report instruments of work ability and functioning, of which 14 were used based on face validity or partially by combining the most relevant questions. The consultations of academic specialists (*n* = 20) improved the 0.1 pilot version’s content. It was also decided that the Abilitator would retain the questions on overall functioning and work ability i.e. Questions B3 and B4 [Additional file 2] throughout the development process. This was to ensure that ESF Priority 5 projects could assess the overall change in work ability and functioning of their clients even if other parts of the Abilitator changed during its development.

We chose the following topics as the main elements of Abilitator 0.1: 1) Work ability and perceived health, 2) Everyday skills, 3) Social functioning and social involvement, 4) Psychological functioning, 5) Cognitive functioning, 6) Physical functioning, and 7) Background information. These topics covered the first three levels of the House of Work Ability and its dimensions of family, close community and society. Abilitator 0.1 contained 57 questions, of which 30 (54%) were taken directly from pre-existing questionnaires [Additional file 1]. The rest were newly-formed questions covering target group-specific topics that had either not been evaluated by a self-assessment method before or for which the formulation of the pre-existing questions did not directly meet the Abilitator criteria; for example, positive question format, equality, generality, and comprehensiveness.

During the development process, the content of the Abilitator was modified twice [Additional file 1]. All the feedback on Abilitator 0.1 and 0.2 was systematically gathered in written format and reviewed in detail by the internal group of experts (Phase 3, 5 and 6, Fig. 1). The suggestions were grouped into similar feedback units and the decisions regarding changes to the questionnaire were made in the internal expert group’s consensus meetings. As a result, 25% of the questions in Abilitator 0.1 remained unchanged, 50% were modified and 25% were removed. The unchanged questions were perceived as feasible for and by the target group and for evaluative purposes. The content or formulation of the questions was changed if: 1) the questions were not perceived as equal, 2) the questions’ original design was not perceived as suitable for the target group, 3) the questions’ original design did not reveal the desired issue precisely enough, 4) the questions required more text to support their comprehension, 5) the questions’ themes were perceived as too narrow or extensive, 6) the questions lacked important areas or response options and 7) the questions had too many or too few response options. The questions removed from Abilitator 0.1 were: 1) not answered as regularly as the others, 2) perceived as repetition, 3) not perceived as appropriate for or by the target population, 4) not perceived as covering the desired aspect, 5) not perceived as equal and 6) too difficult to answer. Nineteen completely new questions were added to Abilitator 0.2. If important issues or sub-issues were completely missing, or if new questions were needed to better suit the target groups’ situation, the removed question was replaced by a new one.

In Abilitator 0.1, the recall period varied from the present to 2 weeks or a month. According to the feedback, this was confusing to both the respondents and the professionals. Therefore, in Abilitator 0.2, the recall period was harmonised to the current situation, except for Section D (Mind) in which the recall period was set as one month. In addition, the scales were harmonised and presented either horizontally or vertically, and the best option was always at the furthest right or at the top, respectively.

When the final Abilitator was created, 60% of the questions in Abilitator 0.2 remained unchanged, 38% were modified and 2% were removed. The unchanged questions were perceived as feasible for and by the target group and for evaluation purposes. The content or formulation of the questions was changed if: 1) the questions required more text to support their comprehension, 2) the order of the questions was not logical within the sections of the questionnaire, 3) the question’s topic was too extensive to answer and needed splitting into two separate questions. Based on the feedback received, we added three new questions to the questionnaire to obtain a broader view of the respondent’s situation. At the end of the development process, the Abilitator contained 84 questions of which 17 were items from existing questionnaires, and 67 were either modifications or completely new items. The content of the personal feedback did not change significantly.

### Information on the Abilitator's content and its use in practice

**Fig. 3:**
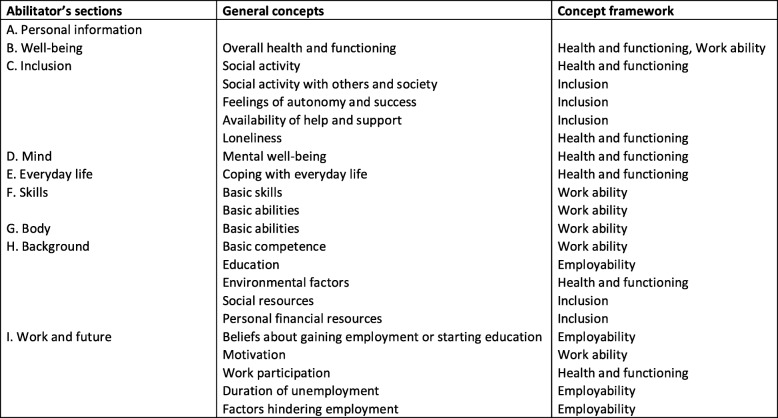
Sections of the Abilitator in relation to its general concepts and concept framework

The Abilitator contains nine sections: A. Personal information, B. Well-being, C. Inclusion, D. Mind, E. Everyday life, F. Skills, G. Body, H. Background information, and I. Work and the Future (Fig. 3). Each section contains 4–14 questions. In Fig. 3, the Abilitator’s sections are further linked to general concepts and the Abilitator’s concept framework presented in Fig. 2. The whole questionnaire is presented in Additional file 2 and can also be accessed online [75].

The interpretation of the results as given in a respondent’s written feedback can be seen in Additional file 3. The feedback is built directly on the response options and has no external benchmark figures. The measure of each section is a summary scale of the selected item. The points received are converted into percentages: the minimum score is 0% and the maximum 100%. The feedback is grouped on the basis of the respondent’s situation per sections B–G: 1) the situation is good, 2) the situation is fairly good, but has some possible challenges and 3) the situation is fairly poor or poor. If the respondent evaluates some items as very poor and others as good, the feedback indicates possible challenges. The Abilitator’s content and its development versions 0.1 and 0.2, the scales, and the ICF codes by question are illustrated in Additional file 1. Another way in which to interpret the results is to do so question by question. The instructions for this are presented in the Abilitator’s user manual, currently only available in Finnish [75].

In practice, the Abilitator can be used in different ways. A service actor working in, for example, employment services can send the client a personal link to the Abilitator via email well before a scheduled appointment. On average the questionnaire takes 15–20 min to complete. The client can complete the questionnaire online independently or with a close person. Another option is that the service actor interviews the client and enters the responses directly into the online version of the Abilitator. A third option is that the service actor either gives or sends the Abilitator questionnaire in paper format to the client. The client then completes the questionnaire and returns it to the service actor, who enters the information into the online version.

The advantage of the online version of the Abilitator is that both the client and the service actor can see the results and personal feedback and prepare for their appointment accordingly. During the appointment, the client and the service actor can discuss the results, and plan targets and actions to improve or sustain the client’s work ability or functioning if necessary. In an ideal situation, they arrange a follow-up appointment during which they evaluate whether these targets have been met.

### Correspondence between the Abilitator’s content and its construct

**Fig. 4:**
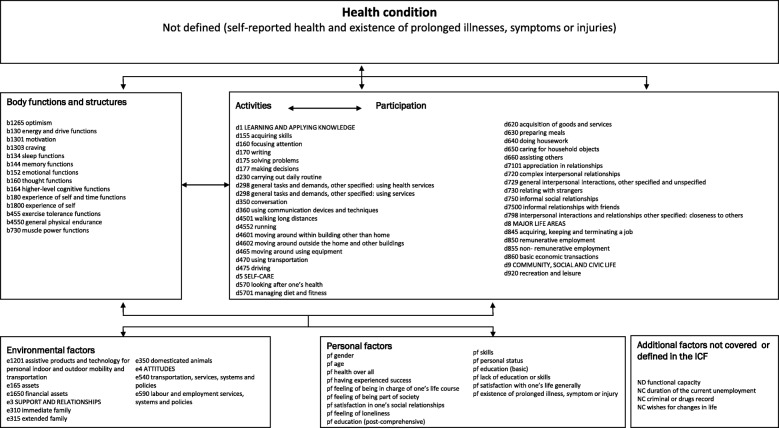
Content of the Abilitator described using the ICF framework

The Abilitator covered 79 ICF codes, of which 14 (18%) described body functions and structures (b), 40 (50%) activities and participation (d), 10 (13%) environmental factors (e) and 15 (19%) personal factors (pf). The ICF codes describing body structures and functions were related to global and specific mental and respiratory system functions. The codes related to activities and participation covered learning and applying knowledge, carrying out general tasks and demands, communication, mobility, self-care, domestic life, interpersonal interactions and relationships, and major life areas. The codes covering environmental factors described products and technology, support, relationships, and attitudes. The correspondence of all the Abilitator’s items to the ICF categories is illustrated in Fig. 4 and Additional file 1.

### Assessment framework for the correspondence between the Abilitator and its construct

The direct equivalence of the Abilitator to the generic set was 4/7 codes (57%); to the brief vocational rehabilitation set, 10/13 codes (77%); and to the minimal environmental set, 1/12 codes (8%). In addition, two d4-category codes of the generic set, one e4-category code of the brief vocational rehabilitation set, and the e3-category codes of the minimal environmental set were indirectly represented in the Abilitator at another category level [Additional file 4].

The direct equivalence of the Abilitator was 5/14 (36%) WAI codes. In addition, similar aspects of four codes were indirectly covered in the e3- and e4-categories. The direct equivalence of the Abilitator was 12/27 (44%) codes of WHODAS 2.0, and there were only minor differences in the codes concerning categories d4 and d5 [Additional file 4].

## Discussion

The purpose of this study was to assess whether the Abilitator covers the relevant aspects needed to assess the overall work ability and functioning of the population in a weak labour market position. The study shows that the Abilitator covers the relevant aspects sufficiently comprehensively to enable this assessment. In terms of the content coverage, the Abilitator covers the basic individual-related elements of work ability, health, functioning, employability, and inclusion. In terms of the content relevance, the sections in the Abilitator: 1) apply to general concepts of work ability and functioning, 2) are relevant to the target population, and 3) are relevant to the purpose of the application of the instrument as a means of evaluation.

As a part of the five-phase content validation process [36, 48], we first specified the construct to be measured and then described the context of the instrument’s use. We used the multidimensional work ability theory [13, 69] and the biopsychosocial model of functioning [15, 58] as a basis for the Abilitator. Work ability and functioning are usually defined in relation to work and health. However, as an unemployed person has no work, the contents related to work ability and functioning in the Abilitator correspond to the general demands of working life (Fig. 2). In addition, when developing the construct, we reviewed the work ability and functioning of the target population as well as the context in which the Abilitator was to be implemented in different services.

Second, we used various expert panels. During the development process, an internal group of experts and several external groups of academic experts, professionals and clients evaluated the contents’ relevance and coverage as well as the questionnaire format. In addition, cognitive interviews were conducted in the target population to improve the relevance and comprehensiveness of the Abilitator. The feedback from the expert panels during the development of the instrument considerably altered the Abilitator’s content, as 80% of the items in the pilot versions were modified.

The expert panels’ role was also important after the Abilitator’s usability and accessibility had been improved. On one hand, the digital format made the Abilitator quick and easy to administer at any phase of the service process. On the other hand, the option of completing the Abilitator on paper was crucial for some service clients. The questions were phrased positively and simply to help the service clients self-report their situation in a neutral way. Multidimensionality and individuality were considered so that both the respondents and the professionals could receive enough information to advance in the most suitable service process. At the same time, the length of the questionnaire was restricted to prevent it becoming too long and heavy for the respondents to answer and the professionals to analyse. The interpretation of the results was made easy for the service clients through short, positively phrased written feedback. For the professionals, the resulting interpretation was made as uncomplicated as possible through educational material and user support.

Third, we considered the information regarding the content of the measurement instrument [36, 48]. The theoretical framework of the Abilitator was described and full details of the self-report questionnaire was provided [Additional file 2] with the interpretation of the results [Additional file 3]. The development of the Abilitator was also described in detail (Fig. 1).

Fourth, to clarify whether the Abilitator corresponded to the construct, we linked it to the ICF (Fig. 4). This revealed that the Abilitator covered 79 ICF codes, which were distributed to five different aspects of health and disability described by the ICF. The health condition aspect was not defined, as the Abilitator does not cover specific health-related disorders or diseases (ICD). Information on self-rated health (Question B2) and the existence of any long-term illness (Question G8) were considered significant for safe interpretation and personal data protection purposes.

Fifth, the Abilitator’s ICF codes were further compared with the three ICF Core Sets most relevant to the target population [Additional file 4]. The areas of functioning missing from the Abilitator were sensation of pain, stress management, and the existence of health services, systems, and policies. Furthermore, the products and technologies described in the ICF minimal environmental Core Set were not fully covered in the Abilitator. However, overall, the Abilitator seemed to adequately cover functioning when compared with the selected ICF Core Sets. If at some point the Abilitator is revised, it might be appropriate to consider whether these missing items should be added to the questionnaire.

There are no gold standard measures of self-assessed work ability and functioning. Therefore, using the ICF framework, we compared the content of the Abilitator with that of two validated, commonly used self-report instruments i.e. the WAI and WHODAS 2.0. The direct ICF correspondence between both instruments and the Abilitator was quite low. This might be because the WAI is directed more toward assessing the work ability and diagnosed ill-health of those who are in employment [76], and the Abilitator focuses more on the aspects of work ability related to the unemployed i.e. individual resources such as self-rated health and functioning, employability, inclusion, and motivation. Although both the Abilitator and the WHODAS 2.0 cover many of the same ICF categories in terms of activities and participation, their contents differ. The WHODAS 2.0 assesses participation in terms of ICF domain activities and participation, whereas the Abilitator also assesses personal and environmental factors.

The Abilitator’s content validation process was based on the framework [48] recommended by the COSMIN panel [49]. Its content validation and development processes are similar to those described in the PROMIS® Instrument Maturity Model [77], which lists the stages of instrument scientific development from conceptualisation through evidence of psychometric properties in multiple diverse populations. According to this model, the Abilitator is now at stage 1: conceptualisation and item pool development are complete. However, some differences to the PROMIS® model must be noted. The Abilitator’s development process contained extensive co-development with professionals, reflecting their views of the work ability, functioning and life situation of the target population. The different sections of the Abilitator represent areas of life which may each contain several latent traits, and the measure of each section is a summary scale of these items. The items in the different sections of the Abilitator are based more on theory and usefulness in practice than on a data-driven approach, where items are selected to reflect a single well-defined latent trait. Therefore, each section of the Abilitator is to be interpreted as a sensible, meaningful combination of items of wide conceptual categories, derived from input of professionals during the development process.

### Strengths and limitations of the study

The first strength of this study is that its structured, long-term, multidimensional development process was combined with extensive co-development. The use different of expert panels led to combining science and practice, which improved the Abilitator’s content, usability and accessibility. The second strength is its clear, well-structured content validation process and its documentation. The third strength relates to the extensive process of linking the Abilitator to ICF, in which the co-operation and work contribution of academic experts was crucial.

The limitations of this study relate to both the Abilitator as a self-report method and the study itself. The first Abilitator-related limitation concerns the interpretation of results. As the assessment is within-subject only and the scales within it are not yet validated as such, the Abilitator cannot yet be used to measure the level of work ability and functioning. On one hand, no benchmark data on the Abilitator are available because the instrument has only been used in the population in a weak labour market position. On the other hand, very strong benchmark data are available for some parts, because the Abilitator contains the same items as the nationally representative population’s health, functioning and welfare surveys [78, 79], including self-rated health and work ability. However, at this point, the Abilitator results may be presented as an approximation of work ability and functioning. The Abilitator should only be used as an indicative instrument, to indicate the respondent’s work ability- and functioning-related resources when the interpretation of the results is the same as the content of the questions.

The second limitation related to the Abilitator is its lack of coverage on work ability factors especially those of work-related specific skills, competence, values and attitudes. If the Abilitator is revised, it might be appropriate to consider whether these missing factors should be added to the questionnaire.

The third Abilitator-related limitation relates to the context of its use. First, the situation in which the Abilitator is applied needs to be considered, that is, 1) in what services the assessment is conducted, 2) how it is applied, 3) what information can be utilised in the context of use, and 4) what kind of actions can be carried out in the services in terms of the results. Furthermore, the process of applying the Abilitator needs to be evaluated, i.e. how well the method sits in the processes carried out in the services, how the method is implemented, and how is it realised. These aspects need to be investigated in future studies.

The first methodological limitation concerns the lack of a systematic review of existing self-report instruments on work ability and functioning. The literature review mainly focused on methods available in Finnish and with free access. Therefore, we may have missed some potential methods during the Abilitator’s initial development.

The second limitation relates to the groups participating in the co-development of the Abilitator, as they were mostly professionals and clients in the ESF Priority 5 projects. This may have led to the exclusion of some relevant views of other professionals working with or belonging to the target population.

We may also have missed some important opinions of professionals and the target group in the co-development workshops. Moreover, only 28 service clients gave direct feedback on the Abilitator’s content. Even though the professionals delivered the views of the service clients to the internal group of experts during the co-development process, their number was low in proportion to the number of professionals (*n* = 700). Therefore, future research on the Abilitator should specifically focus on service clients’ feedback.

Despite these limitations, the Abilitator has the potential to become a generic, feasible, multidimensional self-report instrument for evaluating the work ability and functioning of individuals in a weak labour market position who use different services. The information on work ability and functioning derived from the Abilitator could be used in these services to discuss and work together with individuals towards the most effective service plans. The positive and empowering form of the Abilitator could also support individuals’ agency. For organisations providing these different services, the Abilitator could be an instrument for systematic data collection on work ability and functioning. It has been suggested that different actors producing health and employment services should form closer partnerships and co-operative networks [33]. The most beneficial situation would be one in which the service clients, actors and organisations operate within the same system when assessing work ability and functioning. The Abilitator could be the unified instrument used in different services to strengthen this co-operation when working with the population in a weak labour market position.

## Conclusion

The purpose of this study was to describe the development and assess the content validity of the Abilitator in terms of content relevance and coverage. From a professional perspective, the Abilitator covers the relevant aspects sufficiently comprehensively to be able to assess the overall situation of work ability and functioning of the population in a weak labour market position. Future studies on the Abilitator’s reliability, structural validity, concurrent and predictive validity, and ability to detect changes over time are needed to gain a more comprehensive view of its applicability.

## Supplementary information

**Additional file 1.** The development of the content of the Abilitator, description of the main concept and ICF category for each question [39, 74, 78–88].

**Additional file 2.** The Abilitator questionnaire.

**Additional file 3.** The interpretation of the Abilitator’s results as it is given in a respondent’s written feedback.

**Additional file 4.** The comparison of ICF codes of the Abilitator, ICF Generic Core Set, ICF Vocational Rehabilitation Core Set, ICF Environmental Factors Core Set, The Work Ability Index (WAI) and WHO Disability Assessment Schedule (WHODAS 2.0). The ICF category of personal factors (pf) is not included.

## Data Availability

The raw material for this study not found in the text or the additional files contains qualitative notes from the co-development process. Access to this material can be requested from the corresponding author Miia Wikström, miia.wikstrom@ttl.fi. The raw material from the cognitive interviews can be accessed by contacting Kirsi Unkila, kirsi.unkila@ttl.fi. The ICF codes for WHODAS 2.0 and WAI are openly available on www.terveysportti.fi/dtk/tmi/koti and for the Generic, Vocational Rehabilitation (brief) and Environmental Factors (minimal set) ICF Core Sets on www.icf-core-sets.org, respectively.

## Abbreviations

COSMIN: COnsensus-based Standards for the selection of health Measurement INstruments
ESF: European Social Fund
EU: European Union
FIOH: Finnish Institute of Occupational Health
ICD: International Classification of Diseases and Related Health Problems
ICF: International Classification of Functioning, Disability and Health
pf: ICF category of Personal Factors
PROMIS: Patient-Reported Outcomes Measurement Information System
PROMs: Patient-Reported Outcome Measures
SOLMU: Social Inclusion and the Change of One’s Work Ability and Capacity-project
THL: Finnish Institute for Health and Welfare
WAI: Work Ability Index
WHO: World Health Organization
WHODAS: WHO Disability Assessment Schedule
