# Correction to: Development and content validity of the Abilitator: a self-report questionnaire on work ability and functioning aimed at the population in a weak labour market position

**DOI:** 10.1186/s12889-020-08623-7

**Published:** 2020-04-17

**Authors:** Miia Wikström, Heidi Anttila, Minna Savinainen, Anne Kouvonen, Matti Joensuu

**Affiliations:** 1 Finnish Institute of Occupational Health, Helsinki, Finland; 2 Finnish Institute for Health and Welfare, Helsinki, Finland; 3 Finnish Institute of Occupational Health, Tampere, Finland; 4 Faculty of Social Sciences, University of Helsinki, Helsinki, Finland; 5 Research Institute of Psychology, SWPSSWPS University of Social Sciences and Humanities, Wroclaw, Poland; 6 UKCRC Centre of Excellence for Public Health (Northern Ireland), Queen’s University Belfast, Belfast, UK

**Correction to: BMC Public Health (2020) 20:327**

**https://doi.org/10.1186/s12889-020-8391-8**

It was highlighted that the original article [1] contained an ambiguity in the citation of Reference 2 in the Results section. This Correction article shows the incorrect and correct sentence that includes this reference.

**Incorrect sentence.**

The literature review conducted in Phase 1 (Fig. 1) found eight different theoretical models for work ability [2] and two models for functioning.

**Correct sentence.**

In the literature review conducted in Phase 1 (Fig. 1) we found several different theoretical models for work ability and two models for functioning. In a recent Finnish review [2], eight models of work ability related to rehabilitation were identified. They are briefly described in the following.
