# Supplementary material for: Development and content validity of the Abilitator: a self-report questionnaire on work ability and functioning aimed at the population in a weak labour market position
Source: BMC Public Health. 2020 Mar 14;20:327. doi: 10.1186/s12889-020-8391-8 (PMC7071596; doi:10.1186/s12889-020-8391-8)
Supplement: Supplementary file 1 — Additional file 1. The development of the content of the Abilitator, description of the main concept and ICF category for each question [39, 74, 78–88]. [file 12889_2020_8391_MOESM1_ESM.pdf]

Additional file 1. The development of the content of the Abilitator, description of the main concept and ICF category for each question.

Abilitator 0.1	Abilitator 0.2	Abilitator	Reference	Main concept	ICF category of the main concept
Are you:<br><br>1 Male<br>2 Female	Are you:<br><br>1 Male<br>2 Female<br>3 Other	(A5) Are you:<br><br>1 Male<br>2 Female<br>3 Other<br><br>Scale: Confirmation or agreement	New formulation in Abilitator	Gender	pf
Current age __________ years	Current age ________________ years	(A6) Current age ___________ years<br><br>Scale: Confirmation or agreement	New formulation in Abilitator	Current age in years	pf
How satisfied are you with your life at this moment?<br>Assess your general satisfaction with life.<br><br>5 Very satisfied<br>4 Fairly satisfied<br>3 Not satisfied, but not dissatisfied either<br>2 Fairly dissatisfied<br>1 Very dissatisfied	How satisfied are you with your life at this moment?<br>Assess your general satisfaction with life.<br><br>5 Very satisfied<br>4 Fairly satisfied<br>3 Not satisfied, but not dissatisfied either<br>2 Fairly dissatisfied<br>1 Very dissatisfied	(B1) How satisfied are you with your life at this moment?<br>Assess your general satisfaction with life.<br><br>5 Very satisfied<br>4 Fairly satisfied<br>3 Not satisfied, but not dissatisfied either<br>2 Fairly dissatisfied<br>1 Very dissatisfied<br><br>Scale: Intensity	80. Kurvinen A, Jolkkonen A. Työllisyyden voimavarat mittari Spatia raportteja 3, 2015.<br><br>81. Punakallio A, Lusa S, editors. Eri-ikäisten palomiesten terveys- ja toimintakyky: 13-vuoden seurantatutkimus. Helsinki: Finnish Institute of Occupational Health; 2011. Finnish.	Satisfaction in life	pf
In your opinion, is your health currently:<br>Assess your health as a whole.<br><br>5 Good<br>4 Fairly good<br>3 Average<br>2 Fairly poor<br>1 Poor	In your opinion, is your health currently:<br>Assess your health as a whole.<br><br>5 Good<br>4 Fairly good<br>3 Average<br>2 Fairly poor<br>1 Poor	(B2) In your opinion, is your health currently:<br>Assess your health as a whole.<br><br>5 Good<br>4 Fairly good<br>3 Average<br>2 Fairly poor<br>1 Poor<br><br>Scale: Intensity	79. Koskinen S, Lundqvist A, Ristiluoma N, editors. Health, functional capacity and welfare in Finland in 2011. Report 68. Helsinki: National Institute for Health and Welfare; 2012.	Current health over all	pf

How well do you cope with your everyday activities and tasks? Choose the number that best matches your situation.<br><br><u>0 1 2 3 4 5 6 7 8 9 10</u><br><br>0 = I cope very poorly<br>10 = I cope very well	How well do you cope with your everyday activities and tasks? Choose the number that best matches your situation. Assess your everyday life in general, and how you cope with it.<br><br><u>0 1 2 3 4 5 6 7 8 9 10</u><br><br>0 = I cope very poorly<br>10 = I cope very well	(B3) How well do you cope with your everyday activities and tasks? Choose the number that best matches your situation. Assess your everyday life in general, and how you cope with it.<br><br><u>0 1 2 3 4 5 6 7 8 9 10</u><br><br>0 = I cope very poorly<br>10 = I cope very well<br><br>Scale: Intensity	New question created for Abilitator	General ability to function in everyday life	d230 carrying out daily routine
Let's assume that your work ability would receive a score of 10 points at its best. What score would you give your current work ability?<br><br><u>0 1 2 3 4 5 6 7 8 9 10</u><br><br>0 = Completely unable to work<br>10 = Work ability at its best	Let's assume that your work ability would receive a score of 10 points at its best. What score would you give your current work ability? If you do not currently work, give your assessment in relation to your last job, or the demands of your occupation. If you have no profession, assess your situation in relation to the work you would like to do.<br><br><u>0 1 2 3 4 5 6 7 8 9 10</u><br><br>0 = Completely unable to work<br>10 = Work ability at its best	(B4) Let's assume that your work ability would receive a score of 10 points at its best. What score would you give your current work ability? If you do not currently work, give your assessment in relation to your last job, or the demands of your occupation. If you have no profession, assess your situation in relation to the work you would like to do.<br><br><u>0 1 2 3 4 5 6 7 8 9 10</u><br><br>0 = Completely unable to work<br>10 = Work ability at its best<br><br>Scale: Intensity	39.Tuomi K, Ilmarinen J, Jahkola M, Katajarinne L, Tulkki A. Työkykyindeksi. Työterveyshuolto 19. Helsinki: Finnish Institute of Occupational Health; 1997. Finnish.<br><br>82.WORK ABILITY INDEX ™. Helsinki: Finnish Institute of Occupational Health; 2014.	Ability to work	d850 remunerative employment<br><br>d855 non-remunerative employment

Not in 0.1	Not in 0.2	(B5) How do you feel in relation to work life at the moment? Choose the number that best matches your situation.<br><br>0 1 2 3 4 5 6 7 8 9 10<br><br>0= Work life or employment does not currently apply to me.<br>1-3= I don't have a job. I'm poorly equipped for work life. I need support in order to obtain employment.<br>4-5= I don't have a job, but I am equipped for work life. I may need support in order to obtain employment<br>6-8 = I have a job. I am equipped for work life. I may however need support in order to stay in employment.<br>9-10= I have a job. I am well-equipped to continue in employment.<br><br>Scale: Confirmation or agreement	New question created for Abilitator	Work<br>help and support	d845 acquiring, keeping and terminating a job<br><br>d850 remunerative employment
Do you receive help in practical matters when you need it?<br><br>4= Very often<br>3= Quite often<br>2= Quite rarely<br>1= Very rarely<br>0= Never	Which of the following describe your situation?<br>Choose a number from a scale of 1 = Completely disagree to 5 = Completely agree.<br><br>I get help when I need it<br>1 2 3 4 5	Which of the following describe your situation?<br>Choose a number from a scale of 1 = Completely disagree to 5 = Completely agree.<br><br>(C1) I get help when I need it<br>1 2 3 4 5<br><br>Scale: Intensity	New formulation in Abilitator	Getting help	e3 support and relationships

Not in 0.1	Which of the following describe your situation?<br>Choose a number from a scale of 1 = Completely disagree to 5 = Completely agree.<br><br>I am necessary others<br>1 2 3 4 5	Which of the following describe your situation?<br>Choose a number from a scale of 1 = Completely disagree to 5 = Completely agree.<br><br>(C2) I am needed by others<br>1 2 3 4 5<br><br>Scale: Intensity	New question created for Abilitator	Being needed	d7101 appreciation in relationships
Not in 0.1	Which of the following describe your situation?<br>Choose a number from a scale of 1 = Completely disagree to 5 = Completely agree.<br><br>I am allowed to express my opinions and they are taken into account<br>1 2 3 4 5	Which of the following describe your situation?<br>Choose a number from a scale of 1 = Completely disagree to 5 = Completely agree.<br><br>(C3) I am allowed to express my opinions and they are taken into account 1 2 3 4 5<br><br>Scale: Intensity	New question created for Abilitator	Being heard and to have an affect	e4 attitudes
Not in 0.1	Which of the following describe your situation?<br>Choose a number from a scale of 1 = Completely disagree to 5 = Completely agree.<br><br>I am appreciated 1 2 3 4 5	Which of the following describe your situation?<br>Choose a number from a scale of 1 = Completely disagree to 5 = Completely agree.<br><br>(C4) I am appreciated 1 2 3 4 5<br><br>Scale: Intensity	New question created for Abilitator	Being appreciated	b1800 experience of self
Not in 0.1	Which of the following describe your situation?<br>Choose a number from a scale of 1 = Completely disagree to 5 = Completely agree.<br><br>I have experienced success<br>1 2 3 4 5	Which of the following describe your situation?<br>Choose a number from a scale of 1 = Completely disagree to 5 = Completely agree.<br><br>(C5) I have experienced success<br>1 2 3 4 5<br><br>Scale: Intensity	New question created for Abilitator	Experiences of success	pf

Not in 0.1	Which of the following describe your situation?<br>Choose a number from a scale of 1 = Completely disagree to 5 = Completely agree.<br><br>I am in charge of the course of my life 1 2 3 4 5	Which of the following describe your situation?<br>Choose a number from a scale of 1 = Completely disagree to 5 = Completely agree.<br><br>(C6) I am in charge of the course of my life 1 2 3 4 5<br><br>Scale: Intensity	New question created for Abilitator	Feeling of being in charge of one's life course	pf
Not in 0.1	Which of the following describe your situation?<br>Choose a number from a scale of 1 = Completely disagree to 5 = Completely agree.<br><br>I feel part of society 1 2 3 4 5	Which of the following describe your situation?<br>Choose a number from a scale of 1 = Completely disagree to 5 = Completely agree.<br><br>(C7) I feel part of society 1 2 3 4 5<br><br>Scale: Intensity	New question created for Abilitator	Feeling of being part of society	pf
Not in 0.1	Which of the following describe your situation?<br>Choose a number from a scale of 1 = Completely disagree to 5 = Completely agree.<br><br>I am happy with my relationships 1 2 3 4 5	Which of the following describe your situation?<br>Choose a number from a scale of 1 = Completely disagree to 5 = Completely agree.<br><br>(C8) I am happy with my relationships 1 2 3 4 5<br><br>Scale: Intensity	New question created for Abilitator	Satisfaction in one's social relationships	pf
Do you feel lonely?<br><br>1 Never<br>2 Very seldom<br>3 Sometimes<br>4 Fairly often<br>5 All the time	Do you feel lonely?<br><br>5 Never<br>4 Very seldom<br>3 Sometimes<br>2 Fairly often<br>1 All the time	(C9) Do you feel lonely?<br><br>5 Never<br>4 Very seldom<br>3 Sometimes<br>2 Fairly often<br>1 All the time<br><br>Scale: Frequency	83. Townsend P. The Family Life of Old People. London: Routledge and Kegan Paul; 1957.<br><br>78. Koponen P, Borodulin K, Lundqvist A, Sääksjärvi K, Koskinen S, editors. Health, functional capacity and welfare in Finland – FinHealth 2017 study. Report 4. Helsinki: National Institute for Health and Welfare; 2018.	Feeling of loneliness	pf

Is there a person close to you, with whom you can openly discuss personal issues and problems?<br><br>1 Yes<br>2 No<br>3 I don't know	Do you have someone you can openly talk to about personal issues and problems?<br><br>1 Yes<br>0 No<br>-1 I don't know	(C10) Do you have someone with whom you can openly discuss personal issues and problems?<br><br>1 Yes<br>0 No<br>-1 I don't know<br><br>Scale: Confirmation or agreement	84. Elo A-L, Leppänen A, Lindström K. OSQ: Occupational stress questionnaire: Urser's instructions. Reviews 19. Helsinki: Finnish Institute of Occupational Health; 1992.	Close relationships	e3 support and relationships
Do you have or is there a pet in your household?<br><br>0= Yes<br>1= No	Do you have a pet or animal you care for, that makes you happy?<br><br>1 Yes<br>0 No<br>-1 I don't know<br><br>Scale: Confirmation or agreement	(C11) Do you have a pet or animal you care for, that makes you happy?<br><br>1 Yes<br>0 No<br>-1 I don't know<br><br>Scale: Confirmation or agreement	New question created for Abilitator	Pets	e350 domesticated animals
Do you take part in a club, organization, society, hobby group or spiritual community (for example a sports club, a tenants' committee, a political party, a choir, a congregation)?<br><br>1= No<br>2=Yes, actively<br>3=Yes, occasionally	Do you have hobbies, or something to do that you enjoy?<br><br>1 Yes<br>0 No<br>-1 I don't know	(C12) Do you have hobbies, or something to do that you enjoy?<br><br>1 Yes<br>0 No<br>-1 I don't know<br><br>Scale: Confirmation or agreement	New formulation in Abilitator 0.2 and Abilitator<br><br>In Abilitator 0.1:<br>85.Kaikkonen R, Murto J, Pentala O, Koskela T, Virtala E, Härkänen T, Koskenniemi T, Ahonen J, Vartiainen E, Koskinen S. Alueellisen terveys- ja hyvinvointitutkimuksen perustulokset 2010-2014. Helsinki: National Institute for Health and Welfare; 2014. Finnish.	Hobbies, other leisure time activity	d9 community, social and civic life

Do you spend time with friends or acquaintances?<br><br>1= No<br>2= Yes, less than once a month<br>3= Yes, 1-3 times a month<br>4= Yes, 1-2 times a week<br>5= Yes, 3 times a week or more<br><br>How often are in contact with your friends, relatives or acquaintances, who do not live in the same household as you in the following ways?<br><br>Meeting face-to-face?<br>1= Almost daily<br>2= 1-2 times a week<br>3= 1-3 times a month<br>4= Less than once a month<br>5= Never<br><br>By phone?<br>1= Almost daily<br>2= 1-2 times a week<br>3= 1-3 times a month<br>4= Less than once a month<br>5= Never<br><br>Through the Internet?<br>0= Constantly<br>1= Almost daily<br>2= 1-2 times a week<br>3= 1-3 times a month<br>4= Less than once a month<br>5= Never	How often are you in touch with your friends, relatives or acquaintances who do not live in the same house as you?<br><br>5 Daily or almost daily<br>4 1-2 times a week<br>3 1-3 times a month,<br>2 Less than once a month<br>1 Never	(C13) How often do you meet or are you in contact with friends, relatives or acquaintances, with whom you do not live?<br><br>5 Daily or almost daily<br>4 1-2 times a week<br>3 1-3 times a month,<br>2 Less than once a month<br>1 Never<br><br>Scale: Frequency	New formulation in Abilitator 0.2 and Abilitator<br><br>In Abilitator 0.1:<br>85.Kaikkonen R, Murto J, Pentala O, Koskela T, Virtala E, Härkänen T, Koskenniemi T, Ahonen J, Vartiainen E, Koskinen S. Alueellisen terveys- ja hyvinvointitutkimuksen perustulokset 2010-2014. Helsinki: National Institute for Health and Welfare; 2014. Finnish.	Meeting with people	d750 informal social relationships

(WHODAS 2.0) In the last 30 days, how much difficulty did you have in:	Not in 0.2	Not in Abilitator			
Getting along with people who are close to you?<br><br>1= None<br>2= Mild<br>3= Moderate<br>4= Severe<br>5= Extreme	How well do the following describe your situation?<br>Choose a number from a scale of 1 = Completely disagree to 5 = Completely agree<br><br>I get on well with those close to me<br>1 2 3 4 5	How well do the following describe your situation?<br>Choose a number from a scale of 1 = Completely disagree to 5 = Completely agree<br><br>(C14) I get on well with those close to me 1 2 3 4 5<br><br>Scale: Intensity	New formulation in Abilitator	Getting along with the people closest	d720 complex interpersonal relationships
Maintaining a friendship?<br><br>1= None<br>2= Mild<br>3= Moderate<br>4= Severe<br>5= Extreme	How well do the following describe your situation?<br>Choose a number from a scale of 1 = Completely disagree to 5 = Completely agree<br><br>I find it easy to maintain my friendships 1 2 3 4 5	How well do the following describe your situation?<br>Choose a number from a scale of 1 = Completely disagree to 5 = Completely agree<br><br>(C15) I find it easy to maintain my friendships 1 2 3 4 5<br><br>Scale: Intensity	New formulation in Abilitator	Keeping up relationships	d7500 informal relationships with friends
Making new friends?<br><br>1= None<br>2= Mild<br>3= Moderate<br>4= Severe<br>5= Extreme	How well do the following describe your situation?<br>Choose a number from a scale of 1 = Completely disagree to 5 = Completely agree<br><br>I find it easy to get to know new people 1 2 3 4 5	How well do the following describe your situation?<br>Choose a number from a scale of 1 = Completely disagree to 5 = Completely agree<br><br>(C16) I find it easy to get to know new people 1 2 3 4 5<br><br>Scale: Intensity	New formulation in Abilitator	Getting to know new people and making friends	d7500 informal relationships with friends
Dealing with people you do not know?<br>1= None<br>2= Mild<br>3= Moderate<br>4= Severe<br>5= Extreme	How well do the following describe your situation?<br>Choose a number from a scale of 1 = Completely disagree to 5 = Completely agree<br><br>I find it easy to socialize with people I do not know 1 2 3 4	How well do the following describe your situation?<br>Choose a number from a scale of 1 = Completely disagree to 5 = Completely agree<br><br>(C17) I find it easy to socialize with people I do not know 1 2 3 4<br><br>Scale: Intensity	New formulation in Abilitator	Getting along with strangers	d730 relating with strangers

The following are statements about thoughts and feelings. For each one, choose the response that best describes your situation over the last month.<br><br>I've been feeling optimistic about the future<br><br>1= Never<br>2= Seldom<br>3= Sometimes<br>4= Often<br>5= All the time	The following are statements about thoughts and feelings. For each one, choose the response that best describes your situation over the last month.<br><br>I've been feeling optimistic about the future<br><br>1= Never<br>2= Seldom<br>3= Sometimes<br>4= Often<br>5= All the time	The following are statements about thoughts and feelings. For each one, choose the response that best describes your situation over the last month.<br><br>(D1) I've been feeling optimistic about the future<br><br>1= Never<br>2= Seldom<br>3= Sometimes<br>4= Often<br>5= All the time<br><br>Scale: Frequency	86.Tennant R, Hiller L, Fishwick R, Platt S, Joseph S, Weich S, Parkinson J, Secker J, Stewart-Brown S. The Warwich-Edinburgh Mental Well-Being Scale (WEMWBS): development and UK validation. Health and Quality of Life Outcomes. 2007; 5: 63.	Optimism about the future	b1265 optimism
The following are statements about thoughts and feelings. For each one, choose the response that best describes your situation over the last month.<br><br>I've been feeling useful<br><br>1= Never<br>2= Seldom<br>3= Sometimes<br>4= Often<br>5= All the time	The following are statements about thoughts and feelings. For each one, choose the response that best describes your situation over the last month.<br><br>I've been feeling useful<br><br>1= Never<br>2= Seldom<br>3= Sometimes<br>4= Often<br>5= All the time	The following are statements about thoughts and feelings. For each one, choose the response that best describes your situation over the last month.<br><br>(D2) I've been feeling useful<br><br>1= Never<br>2= Seldom<br>3= Sometimes<br>4= Often<br>5= All the time<br><br>Scale: Frequency	86.Tennant R, Hiller L, Fishwick R, Platt S, Joseph S, Weich S, Parkinson J, Secker J, Stewart-Brown S. The Warwich-Edinburgh Mental Well-Being Scale (WEMWBS): development and UK validation. Health and Quality of Life Outcomes. 2007; 5: 63.	Feeling of being useful to others	b180 experience of self and time functions
The following are statements about thoughts and feelings. For each one, choose the response that best describes your situation over the last month.<br><br>I've been feeling relaxed<br><br>1= Never<br>2= Seldom<br>3= Sometimes<br>4= Often<br>5= All the time	The following are statements about thoughts and feelings. For each one, choose the response that best describes your situation over the last month.<br><br>I've been feeling relaxed<br><br>1= Never<br>2= Seldom<br>3= Sometimes<br>4= Often<br>5= All the time	The following are statements about thoughts and feelings. For each one, choose the response that best describes your situation over the last month.<br><br>(D3) I've been feeling relaxed<br><br>1= Never<br>2= Seldom<br>3= Sometimes<br>4= Often<br>5= All the time<br><br>Scale: Frequency	86.Tennant R, Hiller L, Fishwick R, Platt S, Joseph S, Weich S, Parkinson J, Secker J, Stewart-Brown S. The Warwich-Edinburgh Mental Well-Being Scale (WEMWBS): development and UK validation. Health and Quality of Life Outcomes. 2007; 5: 63.	Feeling relaxed	b130 energy and drive functions

The following are statements about thoughts and feelings. For each one, choose the response that best describes your situation over the last month.<br><br>I've been dealing with problems well<br>I've thought of solutions and considered different options for moving forward.<br><br>1= Never<br>2= Seldom<br>3= Sometimes<br>4= Often<br>5= All the time	The following are statements about thoughts and feelings. For each one, choose the response that best describes your situation over the last month.<br><br>I've been dealing with problems well<br>I've thought of solutions and considered different options for moving forward.<br><br>1= Never<br>2= Seldom<br>3= Sometimes<br>4= Often<br>5= All the time	The following are statements about thoughts and feelings. For each one, choose the response that best describes your situation over the last month.<br><br>(D4) I've been dealing with problems well<br>I've thought of solutions and considered different options for moving forward.<br><br>1= Never<br>2= Seldom<br>3= Sometimes<br>4= Often<br>5= All the time<br><br>Scale: Frequency	86.Tennant R, Hiller L, Fishwick R, Platt S, Joseph S, Weich S, Parkinson J, Secker J, Stewart-Brown S. The Warwick-Edinburgh Mental Well-Being Scale (WEMWBS): development and UK validation. Health and Quality of Life Outcomes. 2007; 5: 63.	Ability to deal with problems	d175 solving problems
The following are statements about thoughts and feelings. For each one, choose the response that best describes your situation over the last month.<br><br>I've been thinking clearly<br>I've been able to separate my feelings and my actions. My thoughts have stayed clear.<br><br>1= Never<br>2= Seldom<br>3= Sometimes<br>4= Often<br>5= All the time	The following are statements about thoughts and feelings. For each one, choose the response that best describes your situation over the last month.<br><br>I've been thinking clearly<br>I've been able to separate my feelings and my actions. My thoughts have stayed clear.<br><br>1= Never<br>2= Seldom<br>3= Sometimes<br>4= Often<br>5= All the time	The following are statements about thoughts and feelings. For each one, choose the response that best describes your situation over the last month.<br><br>(D5) I've been thinking clearly<br>I've been able to separate my feelings and my actions. My thoughts have stayed clear.<br><br>1= Never<br>2= Seldom<br>3= Sometimes<br>4= Often<br>5= All the time<br><br>Scale: Frequency	86.Tennant R, Hiller L, Fishwick R, Platt S, Joseph S, Weich S, Parkinson J, Secker J, Stewart-Brown S. The Warwick-Edinburgh Mental Well-Being Scale (WEMWBS): development and UK validation. Health and Quality of Life Outcomes. 2007; 5: 63.	Clear thoughts	b160 thought functions

The following are statements about thoughts and feelings. For each one, choose the response that best describes your situation over the last month.<br><br>I’ve been feeling close to other people<br><br>1= Never<br>2= Seldom<br>3= Sometimes<br>4= Often<br>5= All the time	The following are statements about thoughts and feelings. For each one, choose the response that best describes your situation over the last month.<br><br>I’ve been feeling close to other people<br><br>1= Never<br>2= Seldom<br>3= Sometimes<br>4= Often<br>5= All the time	The following are statements about thoughts and feelings. For each one, choose the response that best describes your situation over the last month.<br><br>(D6) I’ve been feeling close to other people<br><br>1= Never<br>2= Seldom<br>3= Sometimes<br>4= Often<br>5= All the time<br><br>Scale: Frequency	86.Tennant R, Hiller L, Fishwick R, Platt S, Joseph S, Weich S, Parkinson J, Secker J, Stewart-Brown S. The Warwich-Edinburgh Mental Well-Being Scale (WEMWBS): development and UK validation. Health and Quality of Life Outcomes. 2007; 5: 63.	Feeling of closeness to others	d798 interpersonal interactions and relationships other specified: closeness to others
The following are statements about thoughts and feelings. For each one, choose the response that best describes your situation over the last month.<br><br>I’ve been able to make up my own mind about things<br><br>1= Never<br>2= Seldom<br>3= Sometimes<br>4= Often<br>5= All the time	The following are statements about thoughts and feelings. For each one, choose the response that best describes your situation over the last month.<br><br>I’ve been able to make up my own mind about things<br><br>1= Never<br>2= Seldom<br>3= Sometimes<br>4= Often<br>5= All the time	The following are statements about thoughts and feelings. For each one, choose the response that best describes your situation over the last month.<br><br>(D7) I’ve been able to make up my own mind about things<br><br>1= Never<br>2= Seldom<br>3= Sometimes<br>4= Often<br>5= All the time<br><br>Scale: Frequency	86.Tennant R, Hiller L, Fishwick R, Platt S, Joseph S, Weich S, Parkinson J, Secker J, Stewart-Brown S. The Warwich-Edinburgh Mental Well-Being Scale (WEMWBS): development and UK validation. Health and Quality of Life Outcomes. 2007; 5: 63.	Ability to make decisions about one’s own affairs and issues	d177 making decisions
During the past month have you often been bothered by feeling down, depressed, or hopeless?<br><br>0= No<br>1= Yes	Not in 0.2	Not in Abilitator	74.Arroll B, Khin N, Kerse N. Screening for depression in primary care with two verbally asked questions: cross sectional study. BMJ. 2003, 327;1144-6.		
During the past month have you often been bothered by little interest or pleasure in doing things?<br>0= No<br>1= Yes	Not in 0.2	Not in Abilitator	74.Arroll B, Khin N, Kerse N. Screening for depression in primary care with two verbally asked questions: cross sectional study. BMJ. 2003, 327;1144-6.-6		

Not in 0.1	The following are statements about thoughts and feelings. For each one, choose the response that best describes your situation over the last month.<br><br>I've been able to take the initiative with my personal affairs<br><br>1= Never<br>2= Seldom<br>3= Sometimes<br>4= Often<br>5= All the time	The following are statements about thoughts and feelings. For each one, choose the response that best describes your situation over the last month.<br><br>(D8) I've been able to take the initiative with my personal affairs<br><br>1= Never<br>2= Seldom<br>3= Sometimes<br>4= Often<br>5= All the time<br><br>Scale: Frequency	New question created for Abilitator	Taking initiative with personal affairs	b130 energy and drive functions
Not in 0.1	The following are statements about thoughts and feelings. For each one, choose the response that best describes your situation over the last month.<br><br>I've taken pleasure in things that are important to me<br><br>1= Never<br>2= Seldom<br>3= Sometimes<br>4= Often<br>5= All the time	The following are statements about thoughts and feelings. For each one, choose the response that best describes your situation over the last month.<br><br>(D9) I've taken pleasure in things that are important to me<br><br>1= Never<br>2= Seldom<br>3= Sometimes<br>4= Often<br>5= All the time<br><br>Scale: Frequency	New question created for Abilitator	Ability to take pleasure in things	b152 emotional functions

How well do you cope currently with the following everyday tasks?<br><br>Housework 1 2 3 4<br>1= I have no trouble coping<br>2= I can cope, but I have some difficulties<br>3= I can cope, but it is very difficult for me<br>4= I am unable to cope	How well do you cope with the following everyday tasks?<br>Please answer even if the services in question are not easily available or if the issue is not relevant to you at the moment.<br><br>Housework 1 2 3 4 5<br>For example: cooking, cleaning, laundry<br>1= I am unable to cope<br>2= I have a lot of trouble coping<br>3= have some trouble coping<br>4= I have very little trouble coping<br>5= I cope well	How well do you cope with the following everyday tasks?<br>Please answer even if the services in question are not easily available or if the issue is not relevant to you at the moment.<br><br>(E1) Housework 1 2 3 4 5<br>For example: cooking, cleaning, laundry<br>1= I am unable to cope<br>2= I have a lot of trouble coping<br>3= have some trouble coping<br>4= I have very little trouble coping<br>5= I cope well<br><br>Scale: Intensity	New formulation in Abilitator 0.2 and Abilitator<br><br>In Abilitator 0.1:<br>79. Koskinen S, Lundqvist A, Ristiluoma N, editors. Health, functional capacity and welfare in Finland in 2011. Report 68. Helsinki: National Institute for Health and Welfare; 2012.	Housework	d630 preparing meals<br><br>d640 doing housework
Not in 0.1	How well do you cope with the following everyday tasks?<br>Please answer even if the services in question are not easily available or if the issue is not relevant to you at the moment.<br><br>Shopping 1 2 3 4 5<br>For example: food, clothes, personal hygiene products<br><br>1= I am unable to cope<br>2= I have a lot of trouble coping<br>3= have some trouble coping<br>4= I have very little trouble coping<br>5= I cope well<br><br>Scale: Intensity	How well do you cope with the following everyday tasks?<br>Please answer even if the services in question are not easily available or if the issue is not relevant to you at the moment.<br><br>(E2) Shopping 1 2 3 4 5<br><br>For example: food, clothes, personal hygiene products<br><br>1= I am unable to cope<br>2= I have a lot of trouble coping<br>3= have some trouble coping<br>4= I have very little trouble coping<br>5= I cope well<br><br>Scale: Intensity	New question created for Abilitator	Shopping	d620 acquisition of goods and services

Running errands in a bank, in public services or in a similar situation.<br><br>1= I have no trouble coping<br>2= I can cope, but I have some difficulties<br>3= I can cope, but it is very difficult for me<br>4= I am unable to cope	How well do you cope with the following everyday tasks?<br>Please answer even if the services in question are not easily available or if the issue is not relevant to you at the moment.<br><br>Using public services 1 2 3 4 5 For example: bank, social insurance office, pharmacy, employment office, social services<br><br>1= I am unable to cope<br>2= I have a lot of trouble coping<br>3= have some trouble coping<br>4= I have very little trouble coping<br>5= I cope well	How well do you cope with the following everyday tasks?<br>Please answer even if the services in question are not easily available or if the issue is not relevant to you at the moment.<br><br>(E3) Using public services 1 2 3 4 5<br>For example: bank, social insurance office, pharmacy, employment office, social services<br><br>1= I am unable to cope<br>2= I have a lot of trouble coping<br>3= have some trouble coping<br>4= I have very little trouble coping<br>5= I cope well<br><br>Scale: Intensity	New formulation in Abilitator 0.2 and Abilitator<br><br>In Abilitator 0.1:<br>79. Koskinen S, Lundqvist A, Ristiluoma N, editors. Health, functional capacity and welfare in Finland in 2011. Report 68. Helsinki: National Institute for Health and Welfare; 2012.	Using public services or other services	d298 general tasks and demands, other specified: using services
Using the internet<br><br>1= I have no trouble coping<br>2= I can cope, but I have some difficulties<br>3= I can cope, but it is very difficult for me<br>4= I am unable to cope	How well do you cope with the following everyday tasks?<br>Please answer even if the services in question are not easily available or if the issue is not relevant to you at the moment.<br><br>Using the internet, searching for information 1 2 3 4 5<br>For example: internet banking, filling in forms, consulting timetables<br><br>1= I am unable to cope<br>2= I have a lot of trouble coping<br>3= have some trouble coping<br>4= I have very little trouble coping<br>5= I cope well	How well do you cope with the following everyday tasks?<br>Please answer even if the services in question are not easily available or if the issue is not relevant to you at the moment.<br><br>(E4) Using the internet, searching for information 1 2 3 4 5<br>For example: internet banking, filling in forms, consulting timetables<br><br>1= I am unable to cope<br>2= I have a lot of trouble coping<br>3= have some trouble coping<br>4= I have very little trouble coping<br>5= I cope well<br><br>Scale: Intensity	New formulation in Abilitator 0.2 and Abilitator<br><br>In Abilitator 0.1:<br>79. Koskinen S, Lundqvist A, Ristiluoma N, editors. Health, functional capacity and welfare in Finland in 2011. Report 68. Helsinki: National Institute for Health and Welfare; 2012.	Using the internet	d360 using communication devices and techniques

Not in 0.1	How well do you cope with the following everyday tasks?<br>Please answer even if the services in question are not easily available or if the issue is not relevant to you at the moment.<br><br>Taking care of personal finances<br>1 2 3 4 5<br>For example: budgeting, paying bills on time<br><br>1= I am unable to cope<br>2= I have a lot of trouble coping<br>3= have some trouble coping<br>4= I have very little trouble coping<br>5= I cope well	How well do you cope with the following everyday tasks?<br>Please answer even if the services in question are not easily available or if the issue is not relevant to you at the moment.<br><br>(E5) Taking care of personal finances<br>1 2 3 4 5<br>For example: budgeting, paying bills on time<br><br>1= I am unable to cope<br>2= I have a lot of trouble coping<br>3= have some trouble coping<br>4= I have very little trouble coping<br>5= I cope well<br><br>Scale: Intensity	New question created for Abilitator	Taking care of finances	d860 basic economic transactions
Not in 0.1	How well do you cope with the following everyday tasks?<br>Please answer even if the services in question are not easily available or if the issue is not relevant to you at the moment.<br><br>Looking after your own health and well-being 1 2 3 4 5<br>For example: personal hygiene, dressing, eating, sleeping, taking medication<br><br>1= I am unable to cope<br>2= I have a lot of trouble coping<br>3= have some trouble coping<br>4= I have very little trouble coping<br>5= I cope well<br><br>Scale: Intensity	How well do you cope with the following everyday tasks?<br>Please answer even if the services in question are not easily available or if the issue is not relevant to you at the moment.<br><br>(E6) Looking after your own health and well-being 1 2 3 4 5<br>For example: a balanced diet, exercising, taking your prescribed medication<br><br>1= I am unable to cope<br>2= I have a lot of trouble coping<br>3= have some trouble coping<br>4= I have very little trouble coping<br>5= I cope well<br><br>Scale: Intensity	New question created for Abilitator	Keeping up health or taking care of health issues	d570 looking after one's health

Not in 0.1	Not in 0.2	How well do you cope with the following everyday tasks?<br>Please answer even if the services in question are not easily available or if the issue is not relevant to you at the moment.<br><br>(E7) Maintaining a regular daily routine and sufficient sleep 1 2 3 4 5<br>For example: functioning during the daytime, feeling alert during the day<br><br>1= I am unable to cope<br>2= I have a lot of trouble coping<br>3= have some trouble coping<br>4= I have very little trouble coping<br>5= I cope well<br><br>Scale: Intensity	New question created for Abilitator	Sleep and circadian rhythm	b134 sleep functions
Not in 0.1	Not in 0.2	How well do you cope with the following everyday tasks?<br>Please answer even if the services in question are not easily available or if the issue is not relevant to you at the moment.<br><br>(E8) Taking care of personal hygiene 1 2 3 4 5<br>For example: washing yourself, wearing clean clothes<br><br>1= I am unable to cope<br>2= I have a lot of trouble coping<br>3= have some trouble coping<br>4= I have very little trouble coping<br>5= I cope well<br><br>Scale: Intensity	New question created for Abilitator	Personal hygiene	D5 self-care

Not in 0.1	How well do you cope with the following everyday tasks?<br>Please answer even if the services in question are not easily available or if the issue is not relevant to you at the moment.<br><br>Using health services 1 2 3 4 5<br>For example: doctor's and dentist's appointments, laboratory tests<br><br>1= I am unable to cope<br>2= I have a lot of trouble coping<br>3= have some trouble coping<br>4= I have very little trouble coping<br>5= I cope well	How well do you cope with the following everyday tasks?<br>Please answer even if the services in question are not easily available or if the issue is not relevant to you at the moment.<br><br>(E9) Using health services 1 2 3 4 5<br>For example: doctor's and dentist's appointments, laboratory tests<br><br>1= I am unable to cope<br>2= I have a lot of trouble coping<br>3= have some trouble coping<br>4= I have very little trouble coping<br>5= I cope well<br><br>Scale: Intensity	New question created for Abilitator	Using the health care services when needed	d298 general tasks and demands, other specified: using health services
Using public transportation (for example a train, a bus, a tram, the subway)?<br><br>1= I have no trouble coping<br>2= I can cope, but I have some difficulties<br>3= I can cope, but it is very difficult for me<br>4= I am unable to cope	How well do you cope with the following everyday tasks?<br>Please answer even if the services in question are not easily available or if the issue is not relevant to you at the moment.<br><br>Getting about outside your home 1 2 3 4 5<br>For example: public transport, your own car, taxis, bicycle, on foot<br><br>1= I am unable to cope<br>2= I have a lot of trouble coping<br>3= have some trouble coping<br>4= I have very little trouble coping<br>5= I cope well	How well do you cope with the following everyday tasks?<br>Please answer even if the services in question are not easily available or if the issue is not relevant to you at the moment.<br><br>(E10) Getting from place to place outside your home 1 2 3 4 5<br>For example: public transport, your own car, taxis, bicycle, on foot<br><br>1= I am unable to cope<br>2= I have a lot of trouble coping<br>3= have some trouble coping<br>4= I have very little trouble coping<br>5= I cope well<br><br>Scale: Intensity	New formulation in Abilitator 0.2 and Abilitator<br><br>In Abilitator 0.1:<br>79. Koskinen S, Lundqvist A, Ristiluoma N, editors. Health, functional capacity and welfare in Finland in 2011. Report 68. Helsinki: National Institute for Health and Welfare; 2012.	Getting around and commuting outside of one's home	d4601 moving around within building other than home<br><br>d4602 moving around outside the home and other buildings<br><br>d470 using transportation<br><br>d475 driving

Not in 0.1	How well do you cope with the following everyday tasks?<br>Please answer even if the services in question are not easily available or if the issue is not relevant to you at the moment.<br><br>Looking after others 1 2 3 4 5<br>For example: children, parents and pets<br><br>1= I am unable to cope<br>2= I have a lot of trouble coping<br>3= have some trouble coping<br>4= I have very little trouble coping<br>5= I cope well	How well do you cope with the following everyday tasks?<br>Please answer even if the services in question are not easily available or if the issue is not relevant to you at the moment.<br><br>(E11) Caring for others 1 2 3 4 5<br>For example: children, parents and pets<br><br>1= I am unable to cope<br>2= I have a lot of trouble coping<br>3= have some trouble coping<br>4= I have very little trouble coping<br>5= I cope well<br><br>Scale: Intensity	New question created for Abilitator	Caring for others	d650 caring for household objects<br><br>d660 assisting others
Are you normally able to concentrate on things?<br><br>5 Very well<br>4 Well<br>3 Well enough<br>2 Poorly<br>1 Very poorly	Are you normally able to concentrate on things? For example, reading a book or newspaper, listening to others, filling in forms.<br><br>5 Very well<br>4 Well<br>3 Well enough<br>2 Poorly<br>1 Very poorly	(F1) Are you normally able to concentrate on things? For example, reading a book or newspaper, listening to others, filling in forms.<br><br>5 Very well<br>4 Well<br>3 Well enough<br>2 Poorly<br>1 Very poorly<br><br>Scale: Intensity	79. Koskinen S, Lundqvist A, Ristiluoma N, editors. Health, functional capacity and welfare in Finland in 2011. Report 68. Helsinki: National Institute for Health and Welfare; 2012.	Concentrating	d160 focusing attention
Are you able to take in new knowledge and learn new skills?<br><br>5 Very well<br>4 Well<br>3 Well enough<br>2 Poorly<br>1 Very poorly	Are you able to take in new knowledge and learn new skills?<br><br>5 Very well<br>4 Well<br>3 Well enough<br>2 Poorly<br>1 Very poorly	(F2) Are you able to take in new knowledge and learn new skills?<br><br>5 Very well<br>4 Well<br>3 Well enough<br>2 Poorly<br>1 Very poorly<br><br>Scale: Intensity	79. Koskinen S, Lundqvist A, Ristiluoma N, editors. Health, functional capacity and welfare in Finland in 2011. Report 68. Helsinki: National Institute for Health and Welfare; 2012.	Ability to learn	d155 acquiring skills

How would you currently rate your memory? Is it:<br><br>5 Very good<br>4 Good<br>3 Satisfactory<br>2 Poor<br>1 Very poor	How would you currently rate your memory? Is it:<br><br>5 Very good<br>4 Good<br>3 Satisfactory<br>2 Poor<br>1 Very poor	(F3) How would you currently rate your memory? Is it:<br><br>5 Very good<br>4 Good<br>3 Satisfactory<br>2 Poor<br>1 Very poor<br><br>Scale: Intensity	79. Koskinen S, Lundqvist A, Ristiluoma N, editors. Health, functional capacity and welfare in Finland in 2011. Report 68. Helsinki: National Institute for Health and Welfare; 2012.	Memory functions	b144 memory functions
Not in 0.1	Not in 0.2	(F4) Have you been diagnosed with a learning, concentration or perception difficulty?<br><br>1 Yes<br>0 No<br>2 I don't know<br><br>Scale: Confirmation or agreement	New question created for Abilitator	Diagnosed difficulties	b164 higher-level cognitive functions<br><br>D1 learning and applying knowledge<br><br>d160 focusing attention
Not in 0.1	How well do the following statements reflect how you feel about the future and your skills? Choose a number from a scale of 1 = Completely disagree to 5 = Completely agree<br><br>I feel positive about the future<br>1 2 3 4 5	How well do the following statements reflect how you feel about the future and your skills? Choose a number from a scale of 1 = Completely disagree to 5 = Completely agree<br><br>(F5) I feel positive about the future<br>1 2 3 4 5<br><br>Scale: Intensity	New question created for Abilitator	Attitudes about the future	b1265 optimism

Not in 0.1	How well do the following statements reflect how you feel about the future and your skills? Choose a number from a scale of 1 = Completely disagree to 5 = Completely agree<br><br>I have dreams and hopes for the future 1 2 3 4 5<br>For example: finding a daily routine, sobriety, education, entering work life	How well do the following statements reflect how you feel about the future and your skills? Choose a number from a scale of 1 = Completely disagree to 5 = Completely agree<br><br>(F6) I have dreams and hopes for the future 1 2 3 4 5<br>For example: finding a daily routine, sobriety, education, entering work life<br><br>Scale: Intensity	New question created for Abilitator	Optimisim about the future	b1265 optimism
Not in 0.1	How well do the following statements reflect how you feel about the future and your skills? Choose a number from a scale of 1 = Completely disagree to 5 = Completely agree<br><br>I am ready to make an effort and take action in order to make my dreams come true 1 2 3 4 5	How well do the following statements reflect how you feel about the future and your skills? Choose a number from a scale of 1 = Completely disagree to 5 = Completely agree<br><br>(F7) I am ready to make an effort and take action in order to make my dreams come true 1 2 3 4 5<br><br>Scale: Intensity	New question created for Abilitator	Diligence<br>Determination<br>Motivation	b130 energy and drive functions
Not in 0.1	How well do the following statements reflect how you feel about the future and your skills? Choose a number from a scale of 1 = Completely disagree to 5 = Completely agree<br><br>I have skills that I can use in work life 1 2 3 4 5	How well do the following statements reflect how you feel about the future and your skills? Choose a number from a scale of 1 = Completely disagree to 5 = Completely agree<br><br>(F8) I have skills that I can use in work life 1 2 3 4 5<br><br>Scale: Intensity	New question created for Abilitator	Skills	pf

Not in 0.1	How well do the following statements reflect how you feel about the future and your skills? Choose a number from a scale of 1 = Completely disagree to 5 = Completely agree<br><br>I am able to express myself in different situations<br>1 2 3 4 5<br>e.g. communicate verbally,<br>write my job application, present myself, take part in discussions	How well do the following statements reflect how you feel about the future and your skills? Choose a number from a scale of 1 = Completely disagree to 5 = Completely agree<br><br>(F9) I am able to verbally express myself in different situations<br>1 2 3 4 5<br>For example: express my opinions, take part in conversations<br><br>Scale: Intensity	New question created for Abilitator	Self-expression (verbal)	d350 conversation
Not in 0.1	How well do the following statements reflect how you feel about the future and your skills? Choose a number from a scale of 1 = Completely disagree to 5 = Completely agree<br><br>I am able to express myself in different situations<br>1 2 3 4 5<br>e.g. communicate verbally,<br>write my job application, present myself, take part in discussions	How well do the following statements reflect how you feel about the future and your skills? Choose a number from a scale of 1 = Completely disagree to 5 = Completely agree<br><br>(F10) I am able to express myself in different situations in writing<br>1 2 3 4 5<br>For example: write a job application<br><br>Scale: Intensity	New question created for Abilitator	Self-expression (written)	d170 writing
In your opinion, is your level of physical fitness:<br><br>5 Good<br>4 Fairly good<br>3 Average<br>2 Fairly poor<br>1 Poor	In your opinion, is your level of physical fitness:<br><br>5 Good<br>4 Fairly good<br>3 Average<br>2 Fairly poor<br>1 Poor	(G1) In your opinion, is your level of physical fitness:<br><br>5 Good<br>4 Fairly good<br>3 Average<br>2 Fairly poor<br>1 Poor<br><br>Scale: Confirmation or agreement	87. Aromaa A, Koskinen S, editors. Health and functional capacity in Finland. Baseline results of the Health 2000 health examination survey. Report B3. Helsinki: National Public Health Institute; 2002.	Over all physical condition	b4550 general physical endurance

Not in 0.1	Not in 0.2	(G2) Do you exercise?<br>Exercise includes all leisure-time physical activity, general physical movement or other physical effort that causes breathlessness and sweating.<br><br>5 I exercise at least three times a week<br>4 I exercise once or twice a week<br>3 I exercise, but not every week<br>2 I exercise less than once a month<br>1 I don't exercise<br><br>Scale: Frequency	New question created for Abilitator	Physical activity, frequency	d5701 managing diet and fitness
	Do you regularly use any aids or equipment for moving around,<br>for example, a wheel chair or a walking stick?<br><br>0 Yes<br>1 No	(G3) Do you regularly use any aids or equipment for moving around,<br>for example, a wheel chair or a walking stick?<br><br>0 Yes<br>1 No<br><br>Scale: Confirmation or agreement	New question created for Abilitator	Regular use of aids to move	e1201 assistive products and technology for personal indoor and outdoor mobility and transportation
Are you able to walk about a kilometre without having to rest?<br><br>4 Yes, with no difficulties<br>3 Yes, but with some difficulties<br>2 Yes, but it is very difficult<br>1 I can't do this at all	Are you able to walk about a kilometre without having to rest?<br><br>4 Yes, with no difficulties<br>3 Yes, but with some difficulties<br>2 Yes, but it is very difficult<br>1 I can't do this at all	If your answer was to G3 was No, please proceed to questions G4 and G6<br><br>(G4) Are you able to walk about a kilometre without having to rest?<br><br>4 Yes, with no difficulties<br>3 Yes, but with some difficulties<br>2 Yes, but it is very difficult<br>1 I can't do this at all<br><br>Scale: Intensity	New formulation in Abilitator	Walking, condition of the circulatory system, endurance	d4501 walking long distances

Can you run a reasonably short distance (about a hundred meters)?<br><br>4 Yes, with no difficulties<br>3 Yes, but with some difficulties<br>2 Yes, but it is very difficult<br>1 I can't do this at all	Can you run a reasonably short distance (about a hundred meters)?<br><br>4 Yes, with no difficulties<br>3 Yes, but with some difficulties<br>2 Yes, but it is very difficult<br>1 I can't do this at all	If your answer was to G3 was No, please proceed to questions G4 and G6<br><br>(G6) Can you run a reasonably short distance (about a hundred meters)?<br><br>4 Yes, with no difficulties<br>3 Yes, but with some difficulties<br>2 Yes, but it is very difficult<br>1 I can't do this at all<br><br>Scale: Intensity	85.Kaikkonen R, Murto J, Pentala O, Koskela T, Virtala E, Härkänen T, Koskenniemi T, Ahonen J, Vartiainen E, Koskinen S. Alueellisen terveys- ja hyvinvointitutkimuksen perustulokset 2010-2014. Helsinki: National Institute for Health and Welfare; 2014. Finnish.	Running, muscular functions, muscle reactivity, body coordination, ability to cope in unexpected situations when moving	d4552 running
	If you use a manual wheelchair or other aid, are you able to spin this or walk about a kilometre without having to rest?<br><br>4 Yes, with no difficulties<br>3 Yes, but with some difficulties<br>2 Yes, but it is very difficult<br>1 I can't do this at all	If your answer to G3 was Yes, please proceed to questions G5 and G7<br><br>(G5) If you use a manual wheelchair or other aid, are you able to spin this or walk about a kilometre without having to rest?<br><br>4 Yes, with no difficulties<br>3 Yes, but with some difficulties<br>2 Yes, but it is very difficult<br>1 I can't do this at all<br><br>Scale: Intensity	New question created for Abilitator	Walking or moving using aids, condition of the circulatory system, endurance	d465 moving around using equipment<br><br>b455 exercise tolerance functions
	If you use a manual wheelchair or other aid, are you able to spin this or walk fast for a short distance?<br><br>4 Yes, with no difficulties<br>3 Yes, but with some difficulties<br>2 Yes, but it is very difficult<br>1 I can't do this at all	If your answer to G3 was Yes, please proceed to questions G5 and G7<br><br>(G7) If you use a manual wheelchair or other aid, are you able to spin this or walk fast for a short distance?<br><br>4 Yes, with no difficulties<br>3 Yes, but with some difficulties<br>2 Yes, but it is very difficult<br>1 I can't do this at all<br><br>Scale: Intensity	New question created for Abilitator	Moving fast with aids, ability to cope in unexpected situations when moving	b730 muscle power functions<br><br>d465 moving around using equipment

Do you suffer from a prolonged illness, handicap or injury?<br>0 No<br>1 Yes<br><br>Scale: Confirmation or agreement	Do you have a long-term illness, symptom, or injury?<br>0 No<br>1 Yes	(G8) Do you suffer from one or more prolonged physical or psychological illness, symptom or injury? By prolonged we mean lasting at least six months.<br><br>0 No<br>1 Yes<br><br>Scale: Confirmation or agreement	New formulation in Abilitator	Illness, symptom or injury	pf
If you answered Yes<br>Assess how much of an impediment these illnesses, handicaps or injuries are. Choose the number that best describes the extent of this impediment.<br><br>Leisure-time activities<br>0 1 2 3 4 5 6 7 8 9 10<br><br>0 = no impediment<br>10 = worst possible impediment or huge impediment	If you answered Yes:<br>Assess how much of an impediment these illnesses, symptoms or injuries are. Choose the number that best describes the extent of this impediment.<br><br>Leisure-time activities<br>0 1 2 3 4 5 6 7 8 9 10<br><br>0 = no impediment<br>10 = worst possible impediment or huge impediment	If you answered Yes to G8:<br>Assess how much of an impediment these illnesses, symptoms or injuries are. Choose the number that best describes the extent of this impediment.<br><br>(G9) Leisure-time activities<br>0 1 2 3 4 5 6 7 8 9 10<br><br>0 = no impediment<br>10 = worst possible impediment or huge impediment<br><br>Scale: Intensity	New formulation in Abilitator	Affects of Health impediments on leisure-time	d920 recreation and leisure
If you answered Yes<br>Assess how much of an impediment these illnesses, handicaps or injuries are. Choose the number that best describes the extent of this impediment.<br><br>Housework<br>0 1 2 3 4 5 6 7 8 9 10<br><br>0 = no impediment<br>10 = worst possible impediment or huge impediment	If you answered Yes:<br>Assess how much of an impediment these illnesses, symptoms or injuries are. Choose the number that best describes the extent of this impediment.<br><br>Housework<br>0 1 2 3 4 5 6 7 8 9 10<br><br>0 = no impediment<br>10 = worst possible impediment or huge impediment	If you answered Yes to G8:<br>Assess how much of an impediment these illnesses, symptoms or injuries are. Choose the number that best describes the extent of this impediment.<br><br>(G10) Housework<br>0 1 2 3 4 5 6 7 8 9 10<br><br>0 = no impediment<br>10 = worst possible impediment or huge impediment<br><br>Scale: Intensity	New formulation in Abilitator	Affects of Health impediments on house work	d640 doing housework

If you answered Yes<br>Assess how much of an impediment these illnesses, handicaps or injuries are. Choose the number that best describes the extent of this impediment.<br><br>Work or possible work<br>0 1 2 3 4 5 6 7 8 9 10<br><br>0 = no impediment<br>10 = worst possible impediment or huge impediment	If you answered Yes:<br>Assess how much of an impediment these illnesses, symptoms or injuries are. Choose the number that best describes the extent of this impediment.<br><br>Work or possible work<br>0 1 2 3 4 5 6 7 8 9 10<br><br>0 = no impediment<br>10 = worst possible impediment or huge impediment	If you answered Yes to G8:<br>Assess how much of an impediment these illnesses, symptoms or injuries are. Choose the number that best describes the extent of this impediment.<br><br>(G11) Work or possible work<br>0 1 2 3 4 5 6 7 8 9 10<br><br>0 = no impediment<br>10 = worst possible impediment or huge impediment<br><br>Scale: Intensity	New formulation in Abilitator	Affects of Health impediments on work or possible work	d850 remunerative employment<br><br>d855 non-remunerative employment
Not in 0.1	Not in 0.2	If you answered Yes to G8:<br>Assess how much of an impediment these illnesses, symptoms or injuries are. Choose the number that best describes the extent of this impediment.<br><br>(G12) Personal relationships<br>0 1 2 3 4 5 6 7 8 9 10<br><br>0 = no impediment<br>10 = worst possible impediment or huge impediment<br><br>Scale: Intensity	New question created for Abilitator	Affects of Health impediments on social life	d729 general interpersonal interactions, other specified and unspecified

What is your personal status? You may choose more than one option.<br>Please reply in accordance with your true situation.<br><br>1 I live alone<br>2 I live with my parent or parents<br>3 I am a single parent<br>4 Married or co-habiting, no children<br>5 Married or co-habiting with children<br>6 I live in a household with several other adults<br>7 I live in a group home or an institution<br>8 I don’t have a permanent address	What is your personal status? You may choose more than one option.<br>Please reply in accordance with your true situation.<br><br>1 I live alone<br>2 I live with my parent or parents<br>3 I am a single parent<br>4 I have joint custody of my children<br>5 Married or co-habiting, no children<br>6 Married or co-habiting with children<br>7 I live in a household with several other adults,<br>e.g. house-sharing or student accommodation<br>8 I live in a group home or an institution<br>9 I live in a reception centre<br>10 I live in a sheltered housing unit<br>11 I don’t have a permanent address	(H1) What is your personal status? You may choose more than one option.<br>Please reply in accordance with your true situation.<br><br>1 I live alone<br>2 I live with my parent or parents<br>3 I am a single parent<br>4 I have joint custody of my children<br>5 Married or co-habiting, no children<br>6 Married or co-habiting with children<br>7 I live in a household with several other adults,<br>e.g. house-sharing or student accommodation<br>8 I live in a group home or an institution<br>9 I live in a reception centre<br>10 I live in a sheltered housing unit<br>11 I don’t have a permanent address<br><br>Scale: Confirmation or agreement	New formulation in Abilitator	Personal status	pf
Does the total income of your household cover your costs:<br><br>6 Very easily<br>5 Easily<br>4 Fairly easily<br>3 Fairly poorly<br>2 Poorly<br>1 Very poorly	Does the total income of your household cover your costs:<br><br>6 Very easily<br>5 Easily<br>4 Fairly easily<br>3 Fairly poorly<br>2 Poorly<br>1 Very poorly	(H2) Does the total income of your household cover your costs:<br><br>6 Very easily<br>5 Easily<br>4 Fairly easily<br>3 Fairly poorly<br>2 Poorly<br>1 Very poorly<br><br>Scale: Confirmation or agreement	85.Kaikkonen R, Murto J, Pentala O, Koskela T, Virtala E, Härkänen T, Koskenniemi T, Ahonen J, Vartiainen E, Koskinen S. Alueellisen terveys- ja hyvinvointitutkimuksen perustulokset 2010-2014. Helsinki: National Institute for Health and Welfare; 2014. Finnish.	Total income of household	e1650 financial assets

What is your educational background?<br><br>Basic education:<br>1 Former primary school<br>2 Current comprehensive school, primary school<br>3 I am currently at comprehensive school<br>4 I dropped out of comprehensive school<br>5 I have no basic education	What is your educational background?<br><br>Basic education:<br>1 Comprehensive school, primary school<br>2 I am currently at comprehensive school<br>3 I dropped out of comprehensive school<br>4 I have no basic education	What is your educational background?<br><br>(H3) Basic education:<br>1 Comprehensive school, primary school<br>2 I am currently at comprehensive school<br>3 I dropped out of comprehensive school<br>4 I have no basic education<br><br>Scale: Confirmation or agreement	New formulation in Abilitator	Education (Basic)	pf
Post-comprehensive education:<br>You may choose more than one option.<br><br>1 No education after comprehensive school<br>2 High school/matriculation<br>3 Preparatory education for upper secondary vocational education and training (VALMA) or other such courses (e.g. education for immigrants)<br>4 Course-based vocational training, further vocational qualification module<br>5 Vocational school or college qualification, also competence-based qualification<br>6 Bachelor's degree<br>7 University degree<br>8 Currently in education<br>9 I dropped out of further education	What is your educational background?<br>Post-comprehensive education:<br>You may choose more than one option.<br><br>1 No education after comprehensive school<br>2 High school/matriculation<br>3 Preparatory education for upper secondary vocational education and training (VALMA) or other such courses (e.g. education for immigrants)<br>4 Course-based vocational training, further vocational qualification module<br>5 Vocational school or college qualification, also competence-based qualification<br>6 Bachelor's degree<br>7 Master's degree<br>8 Licentiate or PhD<br>9 I dropped out of further education	What is your educational background?<br><br>(H4) Post-comprehensive education:<br>You may choose more than one option.<br><br>1 No education after comprehensive school<br>2 High school/matriculation<br>3 Preparatory education for upper secondary vocational education and training (VALMA) or other such courses (e.g. education for immigrants)<br>4 Course-based vocational training, further vocational qualification module<br>5 Vocational school or college qualification, also competence-based qualification<br>6 Bachelor's degree<br>7 Master's degree<br>8 Licentiate or PhD<br>9 I dropped out of further education<br><br>Scale: Confirmation or agreement	New formulation in Abilitator	Education (Post-Comprehensive)	pf

Which of the following best describes your current work situation? You may choose more than one option.<br><br>1 Paid employee including work with pay subsidy<br>2 Entrepreneur or farmer<br>3 Self-employed or freelancer<br>4 Work supported by a grant or scholarship<br>5 Non-paid work, for example voluntary or charity work<br>6 Student<br>7 At home (stay-at-home parent or carer)<br>8 Retired (work disability pension, survivor's pension, rehabilitation allowance<br>9 Training, work trial, workshop work, rehabilitative work or similar<br>10 Unemployed	Which of the following best describes your current work situation? You may choose more than one option.<br><br>1 Paid employee (full-time, part-time, work with pay subsidy)<br>2 Entrepreneur or farmer<br>3 Self-employed, freelancer<br>4 Work supported by funding<br>5 Non-paid work, such as voluntary or charity work<br>6 Community service<br>7 Student, apprentice<br>8 At home (stay-at-home parent or carer)<br>9 Retired (work disability pension, partial work disability, rehabilitation allowance or partial rehabilitation allowance, survivor's pension)<br>10 Trainee<br>11 Workshop work, rehabilitative work<br>12 Work trial<br>13 Unemployed (job-seeker at employment office)<br>14 Unemployed (not a job-seeker at unemployment office)	(I1) Which of the following best describes your current work situation? You may choose more than one option.<br><br>1 Trainee<br>2 Workshop work or rehabilitative work<br>3 Work trial<br>4 Unemployed (job-seeker at employment office)<br>5 Unemployed (not a job-seeker at employment office)<br>6 Non-paid work, for example voluntary or charity work<br>7 Community service<br>8 Student or apprentice<br>9 At home (stay-at-home parent or carer)<br>10 On sick leave or partial sick leave<br>11 Retired (work disability pension, partial work disability, rehabilitation allowance or partial rehabilitation allowance, survivor's pension)<br>12 Paid employee (full-time, part-time, work with pay subsidy)<br>13 Entrepreneur or farmer<br>14 Self-employed or freelancer<br><br>Scale: Confirmation or agreement	New formulation in Abilitator	Work status	d8 major life areas

If you are unemployed, how long has your current period of unemployment lasted?<br>If you are in rehabilitation etc., consider the duration of your unemployment before this.<br><br>1 Less than a year<br>2 1–2 years<br>3 3–4 years<br>4 5–10 years<br>5 Over 10 years<br>6 I have never worked in employment<br>7 I am not currently unemployed	How long has your current period of unemployment lasted? If you are in rehabilitation etc., consider the duration of your unemployment before this.<br><br>1 Less than a year<br>2 1-2 years<br>3 3-4 years<br>4 5-10 years<br>5 Over 10 years<br>6 I have never worked in employment<br>7 I am not currently unemployed	(I2) How long has your current period of unemployment lasted?<br>If you are in rehabilitation etc., consider the duration of your unemployment before this.<br><br>1 Less than a year<br>2 1–2 years<br>3 3–4 years<br>4 5–7 years<br>5 8–10 years<br>6 Over 10 years<br>7 I have never worked in employment<br>8 I am not currently unemployed<br><br>Scale: Confirmation or agreement	New formulation in Abilitator	Duration of current unemployment	NC
How difficult do the following make it for you to participate in work life?<br><br>Lack of job opportunities<br><br>1. Not difficult at all<br>2. Slightly difficult<br>3. Rather difficult<br>4. I don't know	How difficult do the following make it for you to participate in work life?<br><br>Lack of job opportunities<br><br>1= Extremely difficult<br>2= Rather difficult<br>3= Somewhat difficult<br>4= Slightly difficult<br>5= Not difficult at all<br>6= I don't know	How difficult do the following make it for you to participate in work life?<br><br>(I3) Lack of job opportunities<br><br>1= Extremely difficult<br>2= Rather difficult<br>3= Somewhat difficult<br>4= Slightly difficult<br>5= Not difficult at all<br>6= I don't know<br><br>Scale: Intensity	New formulation in Abilitator	Job opportunities	e590 labour and employment services, systems and policies
Not in 0.1	Not in 0.2	How difficult do the following make it for you to participate in work life?<br><br>(I4) Commuting difficulties<br>For example: difficult transport connections, long distances.<br><br>1= Extremely difficult<br>2= Rather difficult<br>3= Somewhat difficult<br>4= Slightly difficult<br>5= Not difficult at all<br>6= I don't know<br><br>Scale: Intensity	New question created for Abilitator	Distances, commuting	e540 transportation, services, systems and policies

How difficult do the following make it for you to participate in work life?<br><br>Lack of training and skills<br><br>1. Not difficult at all<br>2. Slightly difficult<br>3. Rather difficult<br>4. I don't know	How difficult do the following make it for you to participate in work life?<br><br>Lack of training and skills<br>For example: language skills, lack of professional qualifications or outdated qualifications<br><br>1= Extremely difficult<br>2= Rather difficult<br>3= Somewhat difficult<br>4= Slightly difficult<br>5= Not difficult at all<br>6= I don't know	How difficult do the following make it for you to participate in work life?<br><br>(I5) Lack of training and skills<br>For example: language skills, lack of professional qualifications or outdated<br>qualifications<br><br>1= Extremely difficult<br>2= Rather difficult<br>3= Somewhat difficult<br>4= Slightly difficult<br>5= Not difficult at all<br>6= I don't know<br><br>Scale: Intensity	New formulation in Abilitator	Lack of education or skills	pf
How difficult do the following make it for you to participate in work life?<br><br>Diminished work motivation or desire to work<br><br>1. Not difficult at all<br>2. Slightly difficult<br>3. Rather difficult<br>4. I don't know	How difficult do the following make it for you to participate in work life?<br><br>Diminished work motivation or desire to work<br><br>1= Extremely difficult<br>2= Rather difficult<br>3= Somewhat difficult<br>4= Slightly difficult<br>5= Not difficult at all<br>6= I don't know	How difficult do the following make it for you to participate in work life?<br><br>(I6) Diminished work motivation or desire to work<br><br>1= Extremely difficult<br>2= Rather difficult<br>3= Somewhat difficult<br>4= Slightly difficult<br>5= Not difficult at all<br>6= I don't know<br><br>Scale: Intensity	New formulation in Abilitator	Motivation to work	b1301 motivation
How difficult do the following make it for you to participate in work life?<br><br>Problems connected to health or functional capacity<br><br>1. Not difficult at all<br>2. Slightly difficult<br>3. Rather difficult<br>4. I don't know	How difficult do the following make it for you to participate in work life?<br><br>Problems connected to health or functional capacity<br><br>1= Extremely difficult<br>2= Rather difficult<br>3= Somewhat difficult<br>4= Slightly difficult<br>5= Not difficult at all<br>6= I don't know	How difficult do the following make it for you to participate in work life?<br><br>(I7) Problems connected to health or functional capacity<br><br>1= Extremely difficult<br>2= Rather difficult<br>3= Somewhat difficult<br>4= Slightly difficult<br>5= Not difficult at all<br>6= I don't know<br><br>Scale: Intensity	New formulation in Abilitator	Health, functional capacity	ND

How difficult do the following make it for you to participate in work life?<br><br>Issues outside work (For example: family, financial situation)<br><br>1. Not difficult at all<br>2. Slightly difficult<br>3. Rather difficult<br>4. I don't know	How difficult do the following make it for you to participate in work life?<br><br>Personal life situation<br>For example: family, relatives, friends, financial situation<br><br>1= Extremely difficult<br>2= Rather difficult<br>3= Somewhat difficult<br>4= Slightly difficult<br>5= Not difficult at all<br>6= I don't know	How difficult do the following make it for you to participate in work life?<br><br>(I8) Personal life situation<br>For example: family, relatives, friends<br><br>1= Extremely difficult<br>2= Rather difficult<br>3= Somewhat difficult<br>4= Slightly difficult<br>5= Not difficult at all<br>6= I don't know<br><br>Scale: Intensity	New formulation in Abilitator	Life situation, relationships	e310 immediate family<br><br>e315 extended family
Not in 0.1	How difficult do the following make it for you to participate in work life?<br><br>Alcohol/drug or other addictions<br><br>1= Extremely difficult<br>2= Rather difficult<br>3= Somewhat difficult<br>4= Slightly difficult<br>5= Not difficult at all<br>6= I don't know	How difficult do the following make it for you to participate in work life?<br><br>(I9) Substance dependence and other addictions<br><br>1= Extremely difficult<br>2= Rather difficult<br>3= Somewhat difficult<br>4= Slightly difficult<br>5= Not difficult at all<br>6= I don't know<br><br>Scale: Intensity	New question created for Abilitator	Factors hindering work life participation, one's own experience	b1303 craving
Not in 0.1	Not in 0.2	How difficult do the following make it for you to participate in work life?<br><br>(I10) Criminal or drugs record<br><br>1= Extremely difficult<br>2= Rather difficult<br>3= Somewhat difficult<br>4= Slightly difficult<br>5= Not difficult at all<br>6= I don't know<br><br>Scale: Intensity	New question created for Abilitator	Factors hindering work life participation, one's own experience	NC

How difficult do the following make it for you to participate in work life?<br><br>Issues outside work (For example: family, financial situation)<br><br>1 Not difficult at all<br>2 Slightly difficult<br>3 Rather difficult<br>4 I don't know	Not in 0.2	How difficult do the following make it for you to participate in work life?<br><br>(I11) Financial situation<br>For example: debts, enforcement orders<br><br>1= Extremely difficult<br>2= Rather difficult<br>3= Somewhat difficult<br>4= Slightly difficult<br>5= Not difficult at all<br>6= I don't know<br><br>Scale: Intensity	New formulation in Abilitator	Factors hindering work life participation, one's own experience	e165 assets
How actively have you applied for work or education in the last four weeks?<br><br>1 Haven't applied at all<br>2 Have applied once<br>3 Have applied 2-3 times<br>4 Have applied more than 3 times			New question in Abilitator 0.1		
	Do you believe that you will find paid work to match your current skills?<br>5 Yes, definitely<br>4 Yes, fairly sure<br>3 Maybe, maybe not<br>2 No<br>1 This question is not relevant to me at the moment		Modified from:<br>88. Kirves K. Perceived employability: Antecedents, trajectories and well-being consequences. PhD thesis. University of Tampere. 2014.		
Do you believe you will find any paid work?<br><br>1 Yes, definitely<br>2 Yes, fairly sure<br>3 Maybe, maybe not<br>4 Unlikely<br>5 No<br>6 This question isn't relevant to me at the moment	Do you believe you will find some other kind of paid work?<br>5 Yes, definitely<br>4 Yes, fairly sure<br>3 Maybe, maybe not<br>2 No<br>1 This question is not relevant to me at the moment	(I12) Do you believe you will find paid work?<br><br>5 Yes, definitely<br>4 Yes, fairly sure<br>3 Maybe, maybe not<br>2 No<br>1 This question is not relevant to me at the moment<br>Scale: Confirmation or agreement	New formulation in Abilitator	Optimism for getting employed, one's own experience	b1265 optimism

Do you believe you will find some other work (for example entrepreneurship, self-employment, freelance)?<br><br>1 Yes, definitely<br>2 Yes, fairly sure<br>3 Maybe, maybe not<br>4 Unlikely<br>5 No<br>6 This question isn't relevant to me at the moment<br><br>Scale: Confirmation or agreement	Not in 0.2	Not in Abilitator	New question in Abilitator 0.1		
Do you believe you will find a meaningful study programme or a training course?<br><br>1 Yes, definitely<br>2 Yes, fairly sure<br>3 Maybe, maybe not<br>4 Unlikely<br>5 No<br>6 This question isn't relevant to me at the moment	Do you believe you will find a meaningful study programme or a training course?<br><br>5 Yes, definitely<br>4 Yes, fairly sure<br>3 Maybe, maybe not<br>2 No<br>1 This question is not relevant to me at the moment	(I13) Do you believe you will find a meaningful study programme or a training course?<br><br>5 Yes, definitely<br>4 Yes, fairly sure<br>3 Maybe, maybe not<br>2 No<br>1 This question is not relevant to me at the moment<br><br>Scale: Confirmation or agreement	New question created for Abilitator	Optimism for getting a suitable education, one's own experience	b1265 optimism
Employment or education is not relevant to me at the moment<br>•	Not in 0.2	Not in Abilitator	New question created for Abilitator 0.1		

Not in 0.1	Which areas of your life do you wish would change?<br>You may choose more than one option.<br><br>1 My physical fitness<br>2 Management of everyday life<br>3 My competence and professional skills<br>4 My emotional well-being<br>5 My personal relationships<br>6 My hobbies and general ability to participate<br>7 My financial situation<br>8 My use of alcohol/drugs or other addictions<br>9 I don't know<br>10 I feel no need for improvements	(I14) Which areas of your life do you wish to change? You may choose more than one option.<br><br>1 My work or employment situation<br>2 My competence and professional skills<br>3 My financial situation<br>4 My health<br>5 My sleep and body clock rhythm<br>6 My diet<br>7 My physical fitness<br>8 Management of everyday life<br>9 My emotional well-being<br>10 My personal relationships<br>11 My hobbies and general ability to participate<br>12 My use of alcohol, drugs or other addictions<br>13 I don't know<br>14 I feel no need for improvements<br><br>Scale: Confirmation or agreement	New question created for Abilitator	Wishes for changes in life	NC
