# Supplementary material for: Development and content validity of the Abilitator: a self-report questionnaire on work ability and functioning aimed at the population in a weak labour market position
Source: BMC Public Health. 2020 Mar 14;20:327. doi: 10.1186/s12889-020-8391-8 (PMC7071596; doi:10.1186/s12889-020-8391-8)
Supplement: Supplementary file 2 — Additional file 2. The Abilitator questionnaire. [file 12889_2020_8391_MOESM2_ESM.pdf]

European Union
European Social Fund
Leverage from the EU 2014–2020
Finnish Institute of Occupational Health

abili tator

# A work ability and functional capacity self-assessment questionnaire

The Abilitator is a voluntary self-assessment questionnaire.
Choose the answer option that best describes your situation at this moment.
Only your service provider or contact worker can connect the results to you.
The answers will be transferred to the Finnish Institute of Occupational Health (FIOH) in such a form that you will not be identified from them.
FIOH will only report on the results of the questionnaire on a group level.

# A. Personal details

(A1) **Personal Abilitator number** (0000)

(A2) **Today's date (ddmmyy)**
(For example: 090917)

(A3) **Project's S number** (S00000)

(A4) **Name of project**

(A5) **Are you:**

- [ ] Male
- [ ] Female
- [ ] Other

(A6) **Current age** ______ **years**

# B. Well-being

(B1) **How satisfied are you with your life at this moment?** Assess your general satisfaction with life.

- [ ] Very satisfied
- [ ] Fairly satisfied
- [ ] Not satisfied, but not dissatisfied either
- [ ] Fairly dissatisfied
- [ ] Very dissatisfied

(B2) **In your opinion, is your health currently:** Assess your health as a whole.

- [ ] Good
- [ ] Fairly good
- [ ] Average
- [ ] Fairly poor
- [ ] Poor

(B3) **How well do you cope with your everyday activities and tasks?** Choose the number that best matches your situation. Assess your everyday life in general, and how you cope with it.

| 0 | 1 | 2 | 3 | 4 | 5 | 6 | 7 | 8 | 9 | 10 |
|---|---|---|---|---|---|---|---|---|---|---|

0 = I cope very poorly

10 = I cope very well

(B4) **Let's assume that your work ability would receive a score of 10 points at its best. What score would you give your current work ability? (0 means that you are currently unable to do any work)** If you do not currently work, give your assessment in relation to your last job, or the demands of your occupation. If you have no profession, assess your situation in relation to the work you would like to do.

| 0 | 1 | 2 | 3 | 4 | 5 | 6 | 7 | 8 | 9 | 10 |
|---|---|---|---|---|---|---|---|---|---|---|

0 = Completely unable to work

10 = Work ability at its best

(B5) **How do you feel in relation to work life at the moment?** Choose the number that best matches your situation.

| 0 | 1 | 2 | 3 | 4 | 5 | 6 | 7 | 8 | 9 | 10 |
|---|---|---|---|---|---|---|---|---|---|---|

| | | | | |
|---|---|---|---|---|
| 0 = Work life or employment does not currently apply to me | 1–3 = I don't have a job. I'm poorly equipped for work life. I need support in order to obtain employment. | 4–5 = I don't have a job, but I am equipped for work life. I may need support in order to obtain employment. | 6–8 = I have a job. I am equipped for work life. I may however need support in order to stay in employment. | 9–10 = I have a job. I am well-equipped to continue in employment. |

# C. Inclusion

**Which of the following describe your situation?**
Choose a number from a scale of 1 = Completely disagree to 5 = Completely agree

| | Completely disagree | | | | Completely agree |
|---|---|---|---|---|---|
| (C1) **I get help when I need it** | 1 | 2 | 3 | 4 | 5 |
| (C2) **I am needed by others** | 1 | 2 | 3 | 4 | 5 |
| (C3) **I am allowed to express my opinions and they are taken into account** | 1 | 2 | 3 | 4 | 5 |
| (C4) **I am appreciated** | 1 | 2 | 3 | 4 | 5 |
| (C5) **I have experienced success** | 1 | 2 | 3 | 4 | 5 |
| (C6) **I am in charge of the course of my life** | 1 | 2 | 3 | 4 | 5 |
| (C7) **I feel part of society** | 1 | 2 | 3 | 4 | 5 |
| (C8) **I am happy with my relationships** | 1 | 2 | 3 | 4 | 5 |

(C9) **Do you feel lonely?**

- ☐ Never
- ☐ Very seldom
- ☐ Sometimes
- ☐ Fairly often
- ☐ All the time

(C10) **Do you have someone with whom you can openly discuss personal issues and problems?**

- ☐ Yes
- ☐ No
- ☐ I don't know

(C11) **Do you have a pet or animal you care for, that makes you happy?**

- [ ] Yes
- [ ] No
- [ ] I don't know

(C12) **Do you have hobbies, or something to do that you enjoy?**

- [ ] Yes
- [ ] No
- [ ] I don't know

(C13) **How often do you meet or are you in contact with friends, relatives or acquaintances, with whom you do not live?**

- [ ] Daily or almost daily
- [ ] 1–2 times a week
- [ ] 1–3 times a month,
- [ ] Less than once a month
- [ ] Never

**How well do the following describe your situation?**
Choose a number from a scale of 1 = Completely disagree to 5 = Completely agree

| | Completely disagree | | | | Completely agree |
|---|---|---|---|---|---|
| (C14) **I get on well with those close to me** | 1 | 2 | 3 | 4 | 5 |
| (C15) **I find it easy to maintain my friendships** | 1 | 2 | 3 | 4 | 5 |
| (C16) **I find it easy to get to know new people** | 1 | 2 | 3 | 4 | 5 |
| (C17) **I find it easy to socialize with people I do not know** | 1 | 2 | 3 | 4 | 5 |

# D. Mind

The following are statements about thoughts and feelings. For each one, choose the response that best describes your situation **over the last month**.

| | Never | Seldom | Some-times | Often | All the time |
|---|---|---|---|---|---|
| (D1) **I've been feeling optimistic about the future** | 1 | 2 | 3 | 4 | 5 |
| (D2) **I've been feeling useful** | 1 | 2 | 3 | 4 | 5 |
| (D3) **I've been feeling relaxed** | 1 | 2 | 3 | 4 | 5 |
| (D4) **I've been dealing with problems well** I've thought of solutions and considered different options for moving forward. | 1 | 2 | 3 | 4 | 5 |
| (D5) **I've been thinking clearly** I 've been able to separate my feelings and my actions. My thoughts have stayed clear. | 1 | 2 | 3 | 4 | 5 |
| (D6) **I've been feeling close to other people** | 1 | 2 | 3 | 4 | 5 |
| (D7) **I've been able to make up my own mind about things** | 1 | 2 | 3 | 4 | 5 |
| (D8) **I've been able to take the initiative with my personal affairs** | 1 | 2 | 3 | 4 | 5 |
| (D9) **I've taken pleasure in things that are important to me** | 1 | 2 | 3 | 4 | 5 |

# E. Everyday life

**How well do you cope with the following everyday tasks?**
**Please answer even if the services in question are not easily available or if the issue is not relevant to you at the moment.**

| | | I am unable to cope | I have a lot of trouble coping | I have some trouble coping | I have very little trouble coping | I cope well |
|---|---|---|---|---|---|---|
| (E1) | **Housework** For example: cooking, cleaning, laundry | 1 | 2 | 3 | 4 | 5 |
| (E2) | **Shopping** For example: food, clothes, personal hygiene products | 1 | 2 | 3 | 4 | 5 |
| (E3) | **Using public services** For example: bank, social insurance office, pharmacy, employment office, social services | 1 | 2 | 3 | 4 | 5 |
| (E4) | **Using the internet, searching for information** For example: internet banking, filling in forms, consulting timetables | 1 | 2 | 3 | 4 | 5 |
| (E5) | **Taking care of personal finances** For example: budgeting, paying bills on time | 1 | 2 | 3 | 4 | 5 |
| (E6) | **Looking after your own health and well-being** For example: a balanced diet, exercising, taking your prescribed medication | 1 | 2 | 3 | 4 | 5 |
| (E7) | **Maintaining a regular daily routine and sufficient sleep** For example: functioning during the daytime, feeling alert during the day | 1 | 2 | 3 | 4 | 5 |
| (E8) | **Taking care of personal hygiene** For example: washing yourself, wearing clean clothes | 1 | 2 | 3 | 4 | 5 |
| (E9) | **Using health services** For example: doctor's and dentist's appointments, laboratory tests | 1 | 2 | 3 | 4 | 5 |
| (E10) | **Getting from place to place outside your home** For example: public transport, your own car, taxis, bicycle, on foot | 1 | 2 | 3 | 4 | 5 |
| (E11) | **Caring for others** For example: children, parents and pets | 1 | 2 | 3 | 4 | 5 |

# F. Skills

(F1) **Are you normally able to concentrate on things?** For example, reading a book or newspaper, listening to others, filling in forms.

- [ ] Very well
- [ ] Well
- [ ] Well enough
- [ ] Poorly
- [ ] Very poorly

(F2) **Are you able to take in new knowledge and learn new skills?**

- [ ] Very well
- [ ] Well
- [ ] Well enough
- [ ] Poorly
- [ ] Very poorly

(F3) **How would you currently rate your memory? Is it:**

- [ ] Very good
- [ ] Good
- [ ] Satisfactory
- [ ] Poor
- [ ] Very poor

(F4) **Have you been diagnosed with a learning, concentration or perception difficulty?**

- [ ] Yes
- [ ] No
- [ ] I don’t know

**How well do the following statements reflect how you feel about the future and your skills?**
Choose a number from a scale of 1 = Completely disagree to 5 = Completely agree

| | Completely disagree | | | | Completely agree |
|---|---|---|---|---|---|
| (F5) **I feel positive about the future** | 1 | 2 | 3 | 4 | 5 |
| (F6) **I have dreams and hopes for the future** For example: finding a daily routine, sobriety, education, entering work life | 1 | 2 | 3 | 4 | 5 |
| (F7) **I am ready to make an effort and take action in order to make my dreams come true** | 1 | 2 | 3 | 4 | 5 |
| (F8) **I have skills that I can use in work life** | 1 | 2 | 3 | 4 | 5 |
| (F9) **I am able to verbally express myself in different situations** For example: express my opinions, take part in conversations | 1 | 2 | 3 | 4 | 5 |
| (F10) **I am able to express myself in different situations in writing.** For example: write a job application | 1 | 2 | 3 | 4 | 5 |

# G. Body

(G1) **In your opinion, is your level of physical fitness:**

- ☐ Good
- ☐ Fairly good
- ☐ Average
- ☐ Fairly poor
- ☐ Poor

(G2) **Do you exercise?**
Exercise includes all leisure-time physical activity, general physical movement or other physical effort that causes breathlessness and sweating.

- ☐ I exercise at least three times a week
- ☐ I exercise once or twice a week
- ☐ I exercise, but not every week
- ☐ I exercise less than once a month
- ☐ I don't exercise

(G3) **Do you regularly use any aids or equipment for moving around, for example a wheel chair or a walking stick?**

☐ Yes ☐ No

**If your answer was No, please proceed to questions G4 and G6**

(G4) **Are you able to walk about a kilometre without having to rest?**

- ☐ Yes, with no difficulties
- ☐ Yes, but with some difficulties
- ☐ Yes, but it is very difficult
- ☐ I can't do this at all

(G6) **Can you run a reasonably short distance (about a hundred meters)?**

- ☐ Yes, with no difficulties
- ☐ Yes, but with some difficulties
- ☐ Yes, but it is very difficult
- ☐ I can't do this at all

**If your answer was Yes, please proceed to questions G5 and G7**

(G5) **If you use a manual wheelchair or other aid, are you able to spin this or walk about a kilometre without having to rest?**

- ☐ Yes, with no difficulties
- ☐ Yes, but with some difficulties
- ☐ Yes, but it is very difficult
- ☐ I can't do this at all

(G7) **If you use a manual wheelchair or other aid, are you able to spin this or walk fast for a short distance?**

- ☐ Yes, with no difficulties
- ☐ Yes, but with some difficulties
- ☐ Yes, but it is very difficult
- ☐ I can't do this at all

(G8) **Do you suffer from one or more prolonged physical or psychological illness, symptom or injury?** By prolonged we mean lasting at least six months.

☐ No
☐ Yes

**If you answered Yes:**

**Assess how much of an impediment these illnesses, symptoms or injuries are. Choose the number that best describes the extent of this impediment**

(G9) **Leisure-time activities**

| 0 | 1 | 2 | 3 | 4 | 5 | 6 | 7 | 8 | 9 | 10 |
|---|---|---|---|---|---|---|---|---|---|---|

0 = no impediment

10 = worst possible impediment or huge impediment

(G10) **Housework**

| 0 | 1 | 2 | 3 | 4 | 5 | 6 | 7 | 8 | 9 | 10 |
|---|---|---|---|---|---|---|---|---|---|---|

0 = no impediment

10 = worst possible impediment or huge impediment

(G11) **Work or possible work**

| 0 | 1 | 2 | 3 | 4 | 5 | 6 | 7 | 8 | 9 | 10 |
|---|---|---|---|---|---|---|---|---|---|---|

0 = no impediment

10 = worst possible impediment or huge impediment

(G12) **Personal relationships**

| 0 | 1 | 2 | 3 | 4 | 5 | 6 | 7 | 8 | 9 | 10 |
|---|---|---|---|---|---|---|---|---|---|---|

0 = no impediment

10 = worst possible impediment or huge impediment

# H. Background information

(H1) **What is your personal status? You may choose more than one option. Please reply in accordance with your true situation.**

- ☐ I live alone
- ☐ I live with my parent or parents
- ☐ I am a single parent
- ☐ I have joint custody of my children
- ☐ Married or co-habiting, no children
- ☐ Married or co-habiting with children
- ☐ I live in a household with several other adults, e.g. house-sharing or student accommodation
- ☐ I live in a group home or an institution
- ☐ I live in a reception centre
- ☐ I live in a sheltered housing unit
- ☐ I don’t have a permanent address

(H2) **Does the total income of your household cover your costs:**

- ☐ Very easily
- ☐ Easily
- ☐ Fairly easily
- ☐ Fairly poorly
- ☐ Poorly
- ☐ Very poorly

**What is your educational background?**

(H3) **Basic education:**

- ☐ Comprehensive school, primary school
- ☐ I am currently at comprehensive school
- ☐ I dropped out of comprehensive school
- ☐ I have no basic education

(H4) **Post-comprehensive education: You may choose more than one option.**

- ☐ No education after comprehensive school
- ☐ High school/matriculation
- ☐ Preparatory education for upper secondary vocational education and training (VALMA) or other such courses (e.g. education for immigrants)
- ☐ Course-based vocational training, further vocational qualification module
- ☐ Vocational school or college qualification, also competence-based qualification
- ☐ Bachelor’s degree
- ☐ Master’s degree
- ☐ Licentiate or PhD
- ☐ I dropped out of further education

# I. Work & the future

(I1) **Which of the following best describes your current work situation? You may choose more than one option.**

- ☐ Trainee
- ☐ Workshop work or rehabilitative work
- ☐ Work trial
- ☐ Unemployed (job-seeker at employment office)
- ☐ Unemployed (not a job-seeker at employment office)
- ☐ Non-paid work, for example voluntary or charity work
- ☐ Community service
- ☐ Student or apprentice
- ☐ At home (stay-at-home parent or carer)
- ☐ On sick leave or partial sick leave
- ☐ Retired (work disability pension, partial work disability, rehabilitation allowance or partial rehabilitation allowance, survivor's pension)
- ☐ Paid employee (full-time, part-time, work with pay subsidy)
- ☐ Entrepreneur or farmer
- ☐ Self-employed or freelancer
- ☐ Work supported by a grant or scholarship

(I2) **How long has your current period of unemployment lasted? If you are in rehabilitation etc., consider the duration of your unemployment before this.**

- ☐ Less than a year
- ☐ 1–2 years
- ☐ 3–4 years
- ☐ 5–7 years
- ☐ 8–10 years
- ☐ Over 10 years
- ☐ I have never worked in employment
- ☐ I am not currently unemployed

**How difficult do the following make it for you to participate in work life?**

| | Extremely difficult | Rather difficult | Somewhat difficult | Slightly difficult | Not difficult at all | I don't know |
|---|---|---|---|---|---|---|
| (I3) **Lack of job opportunities** | 1 | 2 | 3 | 4 | 5 | 6 |
| (I4) **Commuting difficulties** For example: difficult transport connections, long distances. | 1 | 2 | 3 | 4 | 5 | 6 |
| (I5) **Lack of training and skills** For example: language skills, lack of professional qualifications or outdated qualifications | 1 | 2 | 3 | 4 | 5 | 6 |
| (I6) **Diminished work motivation or desire to work** | 1 | 2 | 3 | 4 | 5 | 6 |
| (I7) **Problems connected to health or functional capacity** | 1 | 2 | 3 | 4 | 5 | 6 |
| (I8) **Personal life situation** For example: family, relatives, friends | 1 | 2 | 3 | 4 | 5 | 6 |
| (I9) **Substance dependence and other addictions** | 1 | 2 | 3 | 4 | 5 | 6 |
| (I10) **Criminal or drugs record** | 1 | 2 | 3 | 4 | 5 | 6 |
| (I11) **Financial situation** For example: debts, enforcement orders | 1 | 2 | 3 | 4 | 5 | 6 |

(I12) **Do you believe you will find paid work?**

- ☐ Yes, definitely
- ☐ Yes, fairly sure
- ☐ Maybe, maybe not
- ☐ No
- ☐ This question isn't relevant to me at the moment

(I13) **Do you believe you will find a meaningful study programme or a training course?**

- [ ] Yes, definitely
- [ ] Yes, fairly sure
- [ ] Maybe, maybe not
- [ ] No
- [ ] This question isn't relevant to me at the moment

(I14) **Which areas of your life do you wish to change?**
**You may choose more than one option.**

- [ ] My work or employment situation
- [ ] My competence and professional skills
- [ ] My financial situation
- [ ] My health
- [ ] My sleep and body clock rhythm
- [ ] My diet
- [ ] My physical fitness
- [ ] Management of everyday life
- [ ] My emotional well-being
- [ ] My personal relationships
- [ ] My hobbies and general ability to participate
- [ ] My use of alcohol, drugs or other addictions
- [ ] I don't know
- [ ] I feel no need for improvements
