# Supplementary material for: Development and content validity of the Abilitator: a self-report questionnaire on work ability and functioning aimed at the population in a weak labour market position
Source: BMC Public Health. 2020 Mar 14;20:327. doi: 10.1186/s12889-020-8391-8 (PMC7071596; doi:10.1186/s12889-020-8391-8)
Supplement: Supplementary file 3 — Additional file 3. The interpretation of the Abilitator’s results as it is given in a respondent’s written feedback. [file 12889_2020_8391_MOESM3_ESM.pdf]

Additional file 3. The interpretation of the Abilitator's results as it is given in a respondent's written feedback.

Work Ability & Functioning

Points (sum of options from either B3 and B4)	*Description (only written feedback given)*
0-5	Poor situation
6-7	Fairly poor situation
8-9	Good situation
10	Exellent situation

Inclusion

Points (sum of C1-C8 and C14-C17)	*Given percent value (0-100%)*	*Description*
12	0	Poor situation
13	2	
14	4	
15	6	
16	8	
17	10	
18	13	
19	15	
20	17	
21	19	
22	21	
23	23	
24	25	Fairly poor situation
25	27	
26	29	
27	31	
28	33	
29	35	
30	38	
31	40	
32	42	
33	44	
34	46	
35	48	
36	50	Fairly good situation
37	52	
38	54	
39	56	
40	58	
41	60	
42	63	
43	65	
44	67	
45	69	
46	71	
47	73	
48	75	Good situation
49	77	
50	79	
51	81	
52	83	
53	85	
54	88	
55	90	
56	92	
57	94	
58	96	
59	98	
60	100	

Mind

Points (sum of D1-D9)	*Given percent value (0-100%)*	*Description*
9	0	Poor situation
10	3	
11	6	
12	8	
13	11	
14	14	
15	17	
16	19	
17	22	
18	25	Possible challenges
19	28	
20	31	
21	33	
22	36	
23	39	
24	42	
25	44	
26	47	
27	50	
28	53	
29	56	
30	58	Good situation
31	61	
32	64	
33	67	
34	69	
35	72	
36	75	
37	78	
38	81	
39	83	
40	86	
41	89	
42	92	
43	94	
44	97	
45	100	

Everyday life

Points (sum of E1-E11)	*Given percent value (0-100%)*	*Description*
11	0	Poor situation
12	2	
13	5	
14	7	
15	9	
16	11	
17	14	
18	16	
19	18	
20	20	
21	23	
22	25	Fairly poor situation
23	27	
24	30	
25	32	
26	34	
27	36	
28	39	
29	41	
30	43	
31	45	
32	48	
33	50	Fairly good situation
34	52	
35	55	
36	57	
37	59	
38	61	
39	64	
40	66	
41	68	
42	70	
43	73	
44	75	Good situation
45	77	
46	80	
47	82	
48	84	
49	86	
50	89	
51	91	
52	93	
53	95	
54	98	
55	100	

Skills

Points (sum of F1-F3, E4 and F5-F10)	*Given percent value (0-100%)*	*Description*
10	0	Poor situation
11	3	
12	5	
13	8	
14	10	
15	13	
16	15	
17	18	
18	20	
19	23	
20	25	
21	28	
22	30	
23	33	
24	35	
25	38	
26	40	
27	43	
28	45	
29	48	
30	50	Possible challenges
31	53	
32	55	
33	58	
34	60	
35	63	
36	65	
37	68	
38	70	
39	73	
40	75	Good situation
41	78	
42	80	
43	83	
44	85	
45	88	
46	90	
47	93	
48	95	
49	98	
50	100	

Body

Points (sum of options from G1, G5 and G7 or G1, G4 and G6)	*Given percent value (0-100%)*	*Description*
3	0	Poor situation
4	10	
5	20	
6	30	
7	40	Possible challenges
8	50	
9	60	
10	70	
11	80	
12	90	Good situation
13	100	

Other

The questions not taken into account in the Abilitator's respondent's feedback, but are analysed separately according to the instructions given in the Abilitator user manual
**Personal information:** entirely
**Well-being:** B1,B2, B5
**Inclusion:** C9-C13
**Skills:** F4
**Body:** G2, G9-G12
**Background information:** entirely
**Work & the Future:** entirely
