# Supplementary material for: Development and content validity of the Abilitator: a self-report questionnaire on work ability and functioning aimed at the population in a weak labour market position
Source: BMC Public Health. 2020 Mar 14;20:327. doi: 10.1186/s12889-020-8391-8 (PMC7071596; doi:10.1186/s12889-020-8391-8)
Supplement: Supplementary file 4 — Additional file 4. The comparison of ICF codes of the Abilitator, ICF Generic Core Set, ICF Vocational Rehabilitation Core Set, ICF Environmental Factors Core Set, The Work Ability Index (WAI) and WHO Disability Assessment Schedule (WHODAS 2.0). The ICF category of personal factors (pf) is not included. [file 12889_2020_8391_MOESM4_ESM.pdf]

Additional file 4. The comparison of ICF codes of the Abilitator, ICF Generic Core Set, ICF Vocational Rehabilitation Core Set, ICF Environmental Factors Core Set, The Work Ability Index (WAI) and WHO Disability Assessment Schedule (WHODAS 2.0). The ICF category of personal factors (pf) is not included.

ICF codes combined list	The Abilitator (64 codes, pf not included)	Core Set 1. Generic (7 codes)	Core set 2. Vocational Rehabilitation, brief (13 codes)	Core Set 3. Environmental Factors, minimal set (12 codes)	WAI (14 codes)	WHODAS 2.0 (27 codes)
b126 temperament and personality functions					●	
b1265 optimism	●				●	
b130 energy and drive functions	●	●	●			
b1300 energy level					●	
b1301 motivation	●				●	
b1303 craving	●					
b134 sleep functions	●					
b144 memory functions	●					●
b152 emotional functions	●	●				●
b160 thought functions	●					
b164 higher-level cognitive functions	●		●			
b180 experience of self and time functions	●					
b1800 experience of self	●					
b280 sensation of pain		●				
b455 exercise tolerance functions	●		●			
b4550 general physical endurance	●					
b730 muscle power functions	●					
d1 LEARNING AND APPLYING KNOWLEDGE	●					
d155 acquiring skills	●		●			●
d160 focusing attention	●					●
d170 writing	●					
d175 solving problems	●					●
d177 making decisions	●					
d230 carrying out daily routine	●	●				
d240 handling stress and other psychological demands			●			
d298 general tasks and demands, other specified: using health services	●					
d298 general tasks and demands, other specified: using services	●					
d310 communicating with-receiving spoken messages						●
d350 conversation	●					●
d360 using communication devices and techniques	●					
d4 MOBILITY					●	
d410 changing and maintaining body position						●
d415 maintaining body position						●
d450 walking		●				●
d4501 walking long distances	●					
d455 moving around		●				
d4552 running	●					
d460 moving around in different locations						●
d4601 moving around within building other than home	●					

d4602 moving around outside the home and other buildings	•					
d465 moving around using equipment	•					
d470 using transportation	•					
d475 driving	•					
d5 SELF-CARE	•					
d510 washing oneself						•
d540 dressing						•
d550 eating						•
d570 looking after one's health	•					
d5701 managing diet and fitness	•					
d6 DOMESTIC LIFE						•
d620 acquisition of goods and services	•					
d630 preparing meals	•					
d640 doing housework	•					•
d650 caring for household objects	•					
d660 assisting others	•					
d7101 appreciation in relationships	•					
d720 complex interpersonal relationships	•		•			
d729 general interpersonal interactions, other specified and unspecified	•					
d730 relating with strangers	•					•
d750 informal social relationships	•					•
d7500 informal relationships with friends	•					
d760 family relationships						•
d770 intimate relationships						•
d798 interpersonal interactions and relationships other specified: closeness to others	•					
d8 MAJOR LIFE AREAS	•					
d825 vocational training						•
d820 school education						•
d845 acquiring, keeping and terminating a job	•		•			
d850 remunerative employment	•	•	•		•	•
d855 non-remunerative employment	•		•			
d860 basic economic transactions	•					
d870 economic self-sufficiency						•
d9 COMMUNITY, SOCIAL AND CIVIC LIFE	•					•
d920 recreation and leisure	•					•
d940 human rights						•
e110 products or substances for personal use				•		
e115 products and technology for personal use in daily living				•		
e120 products or substances for personal use indoor and outdoor mobility and transportation				•		

e1201 assistive products and technology for personal indoor and outdoor mobility and transportation	•					
e135 products and technology for employment				•	•	
e150 design, construction and building products and technology of buildings of public use				•		
e155 design, construction and building products and technology of buildings of private use				•		
e165 assets	•					
e1650 financial assets	•					
e2 NATURAL ENVIRONMENT AND HUMAN CHANGES TO ENVIRONMENT					•	
e225 climate				•		
e3 SUPPORT AND RELATIONSHIPS	•					
e310 immediate family	•		•	•	•	
e315 extended family	•				•	
e320 friends				•		
e330 people in positions of authority			•		•	
e335 people in subordinate positions					•	
e355 health professionals				•		
e350 domesticated animals	•					
e4 ATTITUDES	•					
e425 individual attitudes of acquaintances, peers, colleagues, neighbours and community members					•	
e430 individual attitudes of people in positions of authority					•	
e450 individual attitudes of health professionals				•		
e540 transportation, services, systems and policies	•					
e580 health services, systems and policies			•	•		
e590 labour and employment services, systems and policies	•		•			
